# Multi-omics analysis reveals a molecular landscape of the early recurrence and early metastasis in pan-cancer

**DOI:** 10.3389/fgene.2023.1061364

**Published:** 2023-04-20

**Authors:** Dan-ni He, Na Wang, Xiao-Ling Wen, Xu-Hua Li, Yu Guo, Shu-heng Fu, Fei-fan Xiong, Zhe-yu Wu, Xu Zhu, Xiao-ling Gao, Zhen-zhen Wang, Hong-jiu Wang

**Affiliations:** ^1^ Key Laboratory of Tropical Translational Medicine of Ministry of Education, College of Biomedical Information and Engineering, Hainan Medical University, Haikou, China; ^2^ College of Bioinformatics Science and Technology, Harbin Medical University, Harbin, China; ^3^ College of Biomedical Information and Engineering, Hainan Medical University, Haikou, China; ^4^ The Medical Laboratory Center, Hainan General Hospital, Haikou, China

**Keywords:** pan-cancer, multi-omics, early recurrence, early metastasis, potential biomarkers

## Abstract

Cancer remains a formidable challenge in medicine due to its propensity for recurrence and metastasis, which can result in unfavorable treatment outcomes. This challenge is particularly acute for early-stage patients, who may experience recurrence and metastasis without timely detection. Here, we first analyzed the differences in clinical characteristics among the primary tumor, recurrent tumor, and metastatic tumor in different stages of cancer, which may be caused by the molecular level. Moreover, the importance of predicting early cancer recurrence and metastasis is emphasized by survival analyses. Next, we used a multi-omics approach to identify key molecular changes associated with early cancer recurrence and metastasis and discovered that early metastasis in cancer demonstrated a high degree of genomic and cellular heterogeneity. We performed statistical comparisons for each level of omics data including gene expression, mutation, copy number variation, immune cell infiltration, and cell status. Then, various analytical techniques, such as proportional hazard model and Fisher’s exact test, were used to identify specific genes or immune characteristics associated with early cancer recurrence and metastasis. For example, we observed that the overexpression of BPIFB1 and high initial B-cell infiltration levels are linked to early cancer recurrence, while the overexpression or amplification of ANKRD22 and LIPM, mutation of IGHA1 and MUC16, high fibroblast infiltration level, M1 polarization of macrophages, cellular status of DNA repair are all linked to early cancer metastasis. These findings have led us to construct classifiers, and the average area under the curve (AUC) of these classifiers was greater than 0.75 in The Cancer Genome Atlas (TCGA) cancer patients, confirming that the features we identified could be biomarkers for predicting recurrence and metastasis of early cancer. Finally, we identified specific early sensitive targets for targeted therapy and immune checkpoint inhibitor therapy. Once the biomarkers we identified changed, treatment-sensitive targets can be treated accordingly. Our study has comprehensively characterized the multi-omics characteristics and identified a panel of biomarkers of early cancer recurrence and metastasis. Overall, it provides a valuable resource for cancer recurrence and metastasis research and improves our understanding of the underlying mechanisms driving early cancer recurrence and metastasis.

## 1 Introduction

In recent years, cancer has become the third most fatal disease due to its high mortality rate of cancer recurrence and metastasis (Pan et al., 2017; Rueda et al., 2019; Fabisiewicz et al., 2020). As medical technology has improved, early detection and treatment of cancer have become increasingly important (Ni et al., 2014; Pasechnikov et al., 2014; Bronkhorst et al., 2019). Despite this, many early-stage patients still experience recurrence and metastasis that are not easily detected in time (Freeman, 2013; Abbosh et al., 2017). To tackle this problem, adjuvant therapies, such as targeted therapy and immune checkpoint therapy, may be an appropriate strategy for early-stage patients at risk of recurrence and metastasis (Bosman, 1995; Mayekar and Bivona, 2017; Nadal and Bellmunt, 2019; Wang et al., 2019) and manipulating the tumor microenvironment can also help control the tumor and improve prognosis (Aran et al., 2015; Arneth, 2019). However, the usage and timing of adjuvant therapy for early-stage cancer patients is a dilemma since not treating may result in recurrence and metastasis in some patients, while treating may result in overtreatment and significant side effects in others, which can also be harmful to patients. Therefore, it is very necessary and important to identify prognostic factors, which can assess the risk of early recurrence and metastasis, to help patients choose more appropriate therapy (Cianfrocca and Goldstein, 2004; Gouri et al., 2020; Khan and Gerber, 2020).

Recent studies have harnessed omics data to identify molecular features associated with cancer recurrence and metastasis in various types of cancer. For example, based on transcriptomics, 76 genes associated with breast cancer metastasis were identified, all of which had specific expression, and 11 gene markers were used to predict the risk of recurrence in colorectal cancer (Wang et al., 2005; Kim et al., 2019). A whole-genome analysis of 299 breast cancer patients has also revealed that a set of 365 gene mutations that were not present in the primary tumor are commonly discovered in metastasis and that the mutation rate of most genes in recurrence or metastasis samples is higher than that in the primary tumor (Yates et al., 2017). From the perspective of copy number variation (CNV), Hayashi et al. (1995) analyzed mutant-allele-specific amplification in colorectal cancer, and Armin et al. analyzed the genes that were most likely to have copy number variants in bladder urothelial cancer (Soave et al., 2020), verifying that copy number variation was associated with recurrence. Moreover, the tumor microenvironment has also been proven to be a critical factor in cancer recurrence and metastasis, with specific components serving as potential prognostic biomarkers. Zhang et al. (2020) summarized the tumor microenvironment components related to recurrence and metastasis, suggesting that they could be used as prognostic biomarkers. Zhai et al. (2019) identified 11 genes that may play a crucial role in early papillary thyroid cancer recurrence using the weighted gene co-expression network analysis. Additionally, multi-gene analysis scores have been shown to be useful in predicting recurrence and distant metastasis in early breast cancer (Gouri et al., 2020). The findings of these studies offer valuable insights into the factors that contribute to cancer recurrence and metastasis and provide a basis for future research in this field.

However, few studies have explored the early detection of recurrence and metastasis that are often difficult to identify clinically, and the focus has primarily been on a single type of cancer or a specific omics data type. Pan-cancer analysis can exhibit common characteristics among cancers, while comparing early- and late-stage cancer can provide insights into the fundamental mechanisms of cancer development (Mishra et al., 2016; Gerstung et al., 2020). Furthermore, multi-omics approaches can integrate data from different omics to uncover the relationships and impacts on the disease process (Sun and Hu, 2016; Jung et al., 2020; Subramanian et al., 2020). Therefore, the identification of biomarkers related to recurrence and metastasis in early pan-cancer using multi-omics data is crucial. Such biomarkers can aid in predicting which early-stage patients are at risk of developing recurrence and metastasis, enabling timely and effective treatment to improve prognosis.

**FIGURE 1:**
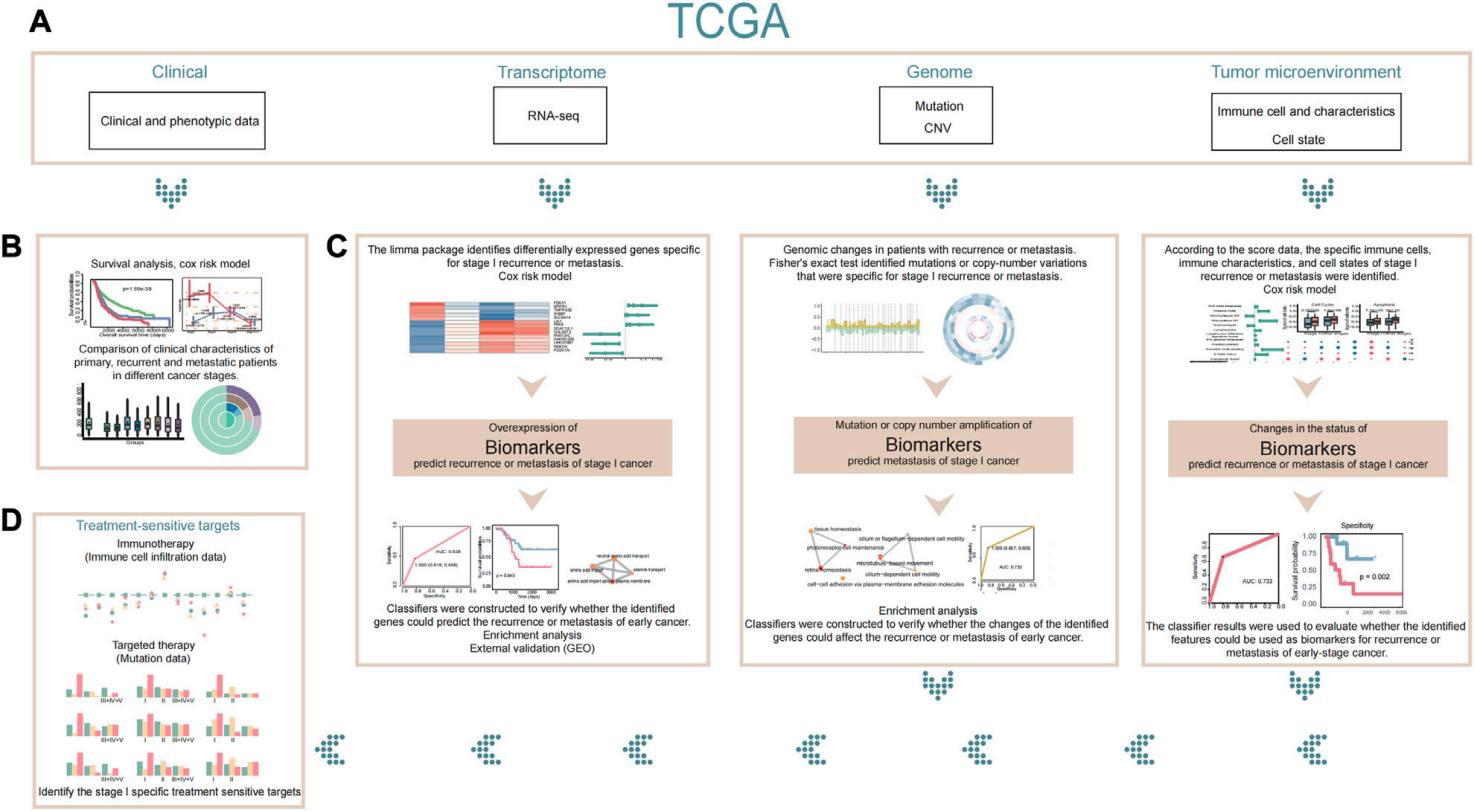
Flowchart. **(A)** Our study is based on clinical phenotypic data, transcriptome data, genomic data, and tumor microenvironment data in TCGA. **(B)** First, we compared the differences in clinical characteristics between patients with stage I recurrence or metastasis and patients with other stages based on clinical data. **(C)** Next, we used transcriptome, genomic, and tumor microenvironment data to explain and analyze the differences in patients with stage I recurrence or metastasis and to identify stage I-specific biomarkers that might predict recurrence or metastasis of early cancer. **(D)** Finally, we identified specific therapeutic sensitive targets for stage I cancer based on existing therapies. If a patient is found to be at high risk of recurrence or metastasis by the biomarkers we identified, appropriate treatment for the patient’s therapeutic sensitive targets may be considered.

Here, we investigated the clinical characteristics of patients with recurrent, metastatic, and primary tumors at different stages to identify specific biomarkers for early recurrence and early metastasis in pan-cancer using multi-omics data. Our results revealed that in genome, immune cell infiltration, and cell status, there were significant differences in the molecular characteristics of early metastasis compared to primary tumors, but no significant differences were observed between early recurrence and primary tumors. Our research also identified possible biomarkers for early recurrence and early metastasis, which could identify early-stage cancer patients with high risk of recurrence and metastasis, and we verified the predictive power of these biomarkers with the constructed classifier results. It was expected to help with clinical treatment and improve prognosis. Additionally, the flow of our entire study is shown in Figure 1.

## 2 Materials and methods

### 2.1 Clinical data

We downloaded the clinical data from the GDC database (https://portal.gdc.cancer.gov/projects), which included information on 12,636 patients such as gender, age, tumor weight, tumor stage, and survival time (days). We eliminated the normal samples or the samples with no stage-related information and the samples with unknown stage-related information. Finally, we obtained the clinical data of 8,342 patients (n = 2,577 patients with stage I cancer, n = 2,370 patients with stage II cancer, n = 2,381 patients with stage III cancer, and n = 1,014 patients with stage IV and stage V cancer).

### 2.2 Gene expression data and copy number variation data

Gene-level RNA-seq expression datasets for 33 types of cancer, which included 11,057 samples and 60,483 genes, were downloaded from UCSC Xena (https://xenabrowser.net/datapages/). We selected the GDC Hub, and then, for each of these 33 cancers, we selected “HTSeq—Counts” for RNA-seq expression data and downloaded the processed data [log_2_ (x+1)].

Meanwhile, gene-level CNV data for 33 cancers of 11,164 samples were also downloaded from UCSC Xena. Similarly, we selected the GDC Hub, and then, for each of these 33 cancers, we selected “GISTIC—focal score by gene” for copy number variations data and downloaded the data. In the CNV data matrix, −1 indicated single-copy deletion and 2 indicated homozygous deletion, which were collectively regarded as copy number deletion. Also, 1 indicated low-level copy number amplification, 2 indicated high-level copy number amplification, and both were collectively regarded as copy number amplification; 0 indicated a diploid normal copy.

### 2.3 External validation data

From the GEO (https://www.ncbi.nlm.nih.gov/geo/) database, we downloaded gene expression and clinical data as external validation data, including GSE31210, GSE37745, GSE44295, and GSE20685. Among them, GSE31210 was the gene expression data of 226 patients, with lung adenocarcinoma at stage I–II, GSE37745 was the gene expression data of 196 non-small lung cancer (NSCLC) cases, with clinical information and long-term follow-up, GSE44295 was the gene expression in primary uveal melanoma cells and normal cell controls of 63 patients, and GSE20685 was the gene expression profiles of 327 breast cancer samples.

### 2.4 Gene mutation data

Somatic mutation data of TCGA patients, with 33 types of cancer, were downloaded from the GDC database. Using the R package of TCGAbiolinks, the specific mutation information of 12,636 patients was obtained from the four somatic mutation platforms, such as Muse, Mutect, SomaticSniper, and VarScan; then, a new mutation information set of 12,636 patients was formed, including mutation type, mutation position, and corresponding amino acid change information, where the union of gene mutation information from four pipelines was adopted and redundant information was removed.

### 2.5 Immune cell infiltration data

We obtained three sets of the immune cell infiltration data in TCGA cancer patients. The first set was downloaded from TIMER (https://cistrome.shinyapps.io/timer/). This dataset involves the infiltrating scores of six immune cells in 11,509 patients with 33 types of cancer, including B cells, CD4 T cells, CD8 T cells, neutrophils, macrophages, and dendritic cells. The other two sets of data were calculated using the R package EPIC (https://github.com/GfellerLab/EPIC) and R package MCPcounter (https://github.com/ebecht/MCPcounter), respectively. The input of MCPcounter was the read count data of gene expression, so we directly used the previously downloaded read count data as input. Also, the input of EPIC was the FPKM data of gene expression, so we downloaded the FPKM data of gene expression (HTSeq-FPKM_UQ) from UCSC Xena. The MCPcounter exported the infiltration scores of 10 immune cells, such as T cells, natural killer cells, and fibroblasts, in 10,906 patients, while EPIC exported the infiltration scores of eight immune cells, such as macrophages, CD4 T cells, and CD8 T cells, in 10,906 patients.

To combine these three sets of data, we normalized [log_2_ (x + 1)] them separately. Next, we combined the three sets of normalized data, and we first intersected the samples to obtain three sets of data of the same samples and then combined the immune cells, in which the same immune cells were averaged, and finally obtained 13 kinds of immune cell infiltration scores of 10,906 patients, including B cells, fibroblasts, CD4 T cells, CD8 T cells, endothelial cells, macrophages, natural killer cells, T cells, monocytes, cytotoxic cells, dendritic cells, neutrophils, and other cells.

### 2.6 T-cell dysregulation signatures obtained in the literature

We obtained 26 genes related to T-cell exhaustion, 25 genes related to T-cell senescence, 292 genes related to T-cell elimination, and 90 genes related to T-cell dysfunction from the article (Jiang et al., 2018; Zhao et al., 2020). All these 433 genes were considered gene signatures related to T-cell dysregulation. Additionally, 160 genes related to T-cell inflammation were obtained from the article (Bao et al., 2020). Thus, a total of 593 genes were used as gene signatures related to T-cell dysregulation.

We downloaded the immune characteristic score of 11,080 patients from literature attachments (Thorsson et al., 2019), including white blood cell data, stromal cell data, and more.

### 2.7 Cancer single-cell functional state

We downloaded cancer cell status data from the CancerSEA (http://biocc.hrbmu.edu.cn/CancerSEA/home.jsp) database, which involved 14 functional cell statuses of 41,900 single cancer cells of 25 cancer types. Additionally, we also downloaded a total of 1,574 genes related to various functional cell states from the CancerSEA database.

### 2.8 Ligand–receptor-related genes

We downloaded the data of ligand–receptor interaction pairs from the CellTalkDB (http://tcm.zju.edu.cn/celltalkdb/) database. In total, there were 3,398 ligand–receptor pairs, including 815 ligand genes and 780 receptor genes.

### 2.9 Therapy-related genes

From the OncoKB database (Chakravarty et al., JCO PO 2017), we obtained 158 genes and their information related to targeted therapy.

Immune checkpoint proteins were downloaded from the Sino Biological database. The genes corresponding to these proteins and their information related to immune checkpoint therapy were obtained from the GeneCards database. Finally, we obtained 49 immune checkpoint-related genes, including 25 immune checkpoint-related genes in T cells and 24 immune checkpoint-related genes in tumor cells.

### 2.10 Survival analysis

We used the Kaplan–Meier curve and the log-rank test to estimate the survival probabilities and test the differences in the survival rate among patient subgroups. Differences were considered significant if the two-sided *p*-value was less than 0.05. These survival analyses were conducted using the “survival” package in the R environment.

### 2.11 Identification of differentially expressed genes

We used the limma package in R to analyze the differential expression of genes in 33 cancer patients, and the input data were the read count data. We first de-log-processed the read count data to obtain the raw count data, which were then normalized using the voom method in the limma package. The voom method normalized the read counts and applied a variance stabilizing transformation to the data, which allowed for the use of linear modeling methods that assumed normally distributed data. Next, we used the limma package to calculate the fold-change and *p*-value for each gene in primary and recurrent tumors or primary and metastatic tumors of different cancer stages. Genes with a fold-change value greater than 2 or less than −2 and a *p*-value less than 0.05 were considered to be differentially expressed.

We further performed multiple testing corrections using the Benjamini–Hochberg method to control the false discovery rate (FDR). Genes with an adjusted *p*-value less than 0.05 were considered to be significantly differentially expressed.

### 2.12 Enrichment analysis of identified genes

To investigate the biological pathways and functions associated with the genes identified in our analysis, we utilized the R package clusterProfiler and org.Hs.eg.db. Specifically, we used the enrichGO and enrichKEGG functions to perform Gene Ontology (GO) and Kyoto Encyclopedia of Genes and Genomes (KEGG) pathway enrichment analyses, respectively. This allowed us to identify the enriched GO terms and KEGG pathways associated with the identified genes.

Additionally, we used the emapplot function from the clusterProfiler package to create an enrichment map that visually represents the relationship between enriched pathways. This provided a comprehensive view of different biological processes that may be involved in the development and progression of cancer. Finally, we analyzed and interpreted the results of the enrichment analysis to gain insights into the underlying mechanisms and potential therapeutic targets associated with the identified genes.

### 2.13 Assessment of predictors for survival and tumor occurrences using Cox proportional hazard regression analysis

We performed a Cox proportional hazard regression analysis to assess the impact of several factors on survival and occurrences of new tumors. We calculated HRs and 95% confidence intervals (CIs) for each predictor variable and used forest plots to present the results. The HR represents the risk or hazard of an event occurring in the presence of the predictor variable as compared to its absence. Variables with an HR greater than 1 and a *p*-value less than 0.05 were considered to be associated with poor prognosis. Each predictor variable was represented by a separate row in the plot, and HRs and 95% CIs were plotted as a horizontal line with a circular marker. The location of the circular marker on the horizontal line indicated the point estimate of the HR, while the length of the horizontal line represents the 95% CI.

### 2.14 Statistics analysis of gene mutations and copy number variations in association with recurrence and metastasis

To investigate the association between gene mutations or copy number variations and recurrence or metastasis, we employed Fisher’s exact test. For each gene, we calculated four values based on samples from different cancer stages: the number of samples with no recurrence (or no metastasis) and no gene mutations, the number of samples with no recurrence (or no metastasis) and gene mutations, the number of samples with recurrence (or metastasis) and no gene mutations, and the number of samples with recurrence (or metastasis) and gene mutations. We then used these four values as input for Fisher’s exact test, which calculated a *p*-value for each gene. Similarly, for copy number variations, we used the same four values as input, but we use the number of samples with or without a copy number variation, instead of having or not having a gene mutation. A *p*-value less than 0.05 was considered statistically significant and indicated that mutations or copy number variations in that gene were associated with recurrence (or metastasis).

### 2.15 Classifier construction for predicting recurrence and metastasis

To evaluate the performance of the identified biomarkers in predicting recurrence and metastasis, we conducted a machine learning analysis. For addressing the imbalance between the number of primary and recurrent/metastatic samples in stage I, we randomly selected the same number as recurrence/metastasis samples from primary tumor samples. We then combined these samples with all the samples from the recurrence/metastasis group and randomly split them into training and test sets, with 80% of the samples in the training set and 20% in the test set. We performed this procedure separately for recurrence and metastasis samples.

Based on the identified biomarkers, we constructed classifiers using support vector machine (SVM), k-nearest neighbor, and random forest algorithms to predict the recurrence/metastasis status of the samples in the test dataset. We then calculated the specificity and sensitivity of each classifier using the actual recurrence/metastasis status of patients. Finally, we plotted receiver operating characteristic (ROC) curves to evaluate the performance of each classifier. The area under the curve (AUC) was calculated with a value closer to 1, indicating better classifier performance.

### 2.16 Cell state analysis using GSVA in tumor samples

To evaluate differences in the cell status between primary and recurrent or metastatic tumors at different cancer stages, we used the R package gene set variation analysis (GSVA). First, we obtained a set of cell state-related genes from the CancerSEA database. This gene set included genes that were known to be associated with various cell states, such as quiescent cells, activated fibroblasts, and immune cells. Next, we used the GSVA function in the GSVA package to calculate the score of the corresponding cell state for each sample with the gene expression data. This allowed us to obtain a quantitative measure of the degree to which each sample was associated with each of the 14 cell states included in the gene set. Finally, we obtained the enrichment scores of all 14 kinds of cell states for each sample. These scores provided a comprehensive view of the extent to which each cell state was represented in the gene expression profile of each tumor sample.

### 2.17 R script

We uploaded all the code and some of the data for this study to GitHub (https://github.com/bioWzz/Multi-omics-profiling-reveals-characterization-of-the-early- recurrence-and-early-metastasis-in-pan-c). All the codes were in the .txt file called multi-omics profiling reveals characterization of the early recurrence and early metastasis in pan-cancer. The rest was part of the input or output data.

## 3 Results

### 3.1 Significant association between early recurrence, metastasis, and negative prognosis of cancer

**FIGURE 2:**
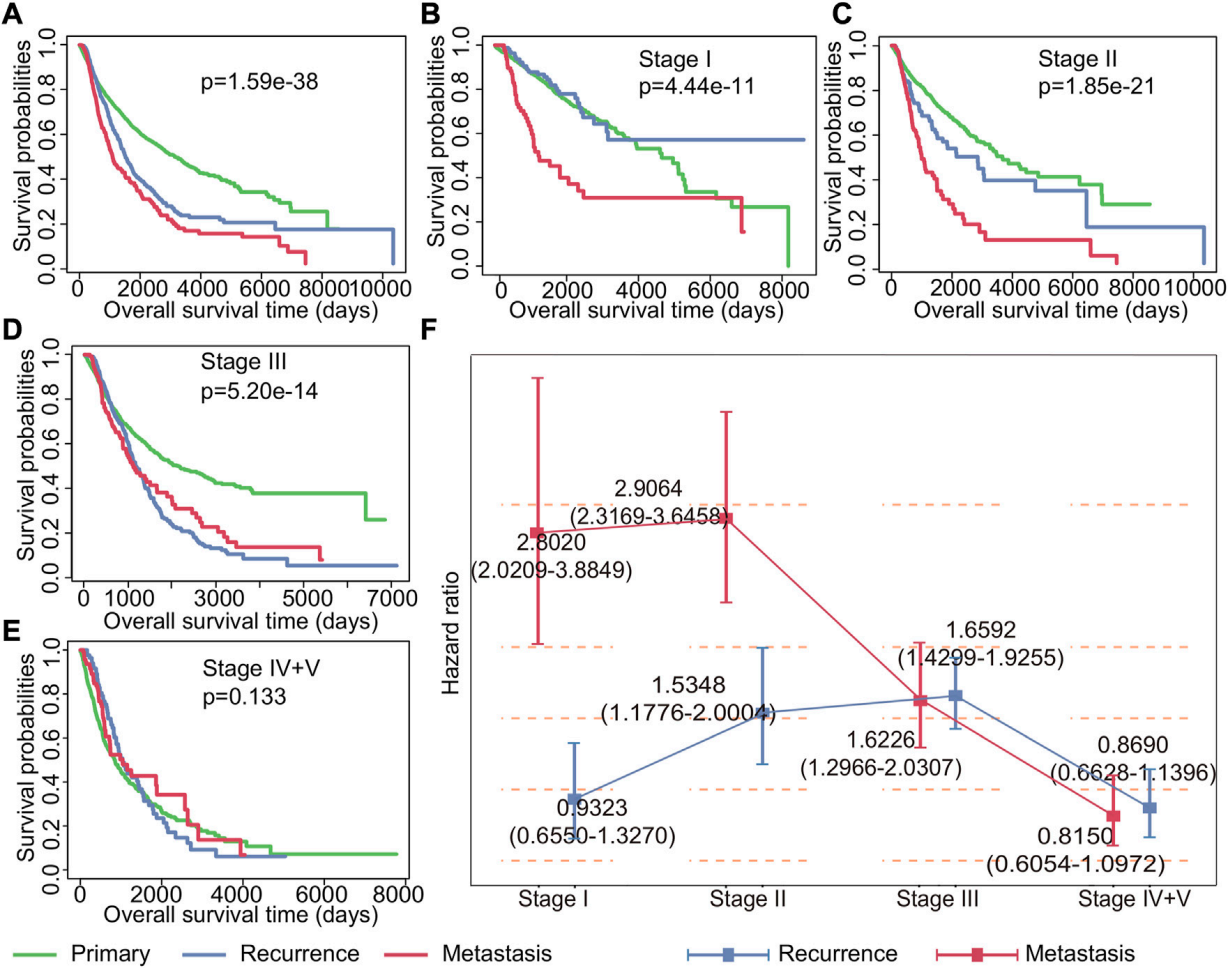
Impact of recurrence and metastasis on patients’ survival in various stages of cancer. **(A)** Kaplan–Meier survival curves of all patients with primary, recurrent, and metastatic tumors. **(B–E)** Kaplan–Meier survival curves of patients with primary tumors, recurrent tumors, and metastatic tumors in stages I, II, III, and IV + V. **(F)** Cox regression analysis of the impact of recurrence and metastasis on each stage patients’ survival. The point represents the risk ratio, and the line segment represents the 95% confidence interval.

Understanding the impact of recurrent and metastatic tumors on patient survival is crucial for improving cancer management strategies. To this end, we conducted a comprehensive analysis of the impact of recurrent and metastatic tumors on survival. Our findings indicated that both recurrence and metastasis have a significant impact on the prognosis for cancer at all stages, with metastasis having a greater impact than recurrence (*p* = 1.59*e^−38^, Figure 2A). Further analysis of patient survival at different stages revealed that recurrence was not a significant risk factor for patients in stage I (*p* = 0.3714224), while metastasis was observed to be a relatively important factor affecting survival (Figure 2B). For patients with stage II (*p* = 1.85*e^−21^) and stage III (*p* = 5.20*e^−14^) cancer, the Kaplan–Meier survival curves showed that both recurrence and metastasis impacted the prognosis (Figures 2C,D). Notably, our results indicated that neither recurrence nor metastasis had any significant impact on the survival of patients in stage IV and V (*p* = 0.133) (Figure 2E).

To further study the impact of recurrence and metastasis on patient survival, we used the Cox proportional hazard regression to investigate the risk of recurrence and metastasis tumors for patient survival. Our findings, as shown in Figure 2F, revealed that the effects of recurrence and metastasis on survival varied depending on the tumor stage. Specifically, the impact of recurrence on poor prognosis increased with the advancement of tumor stages, with the exception of stage IV and stage V (HR for stage I: 0.9323 (0.6550–1.3270), HR for stage II: 1.5348 (1.1776–2.0004), HR for stage III: 1.6592 (1.4299–1.9255), and HR for stage IV and V: 0.8690 (0.6628–1.1396)). Conversely, the impact of metastasis on poor prognosis decreases with the development of tumor except for stage II (HR for stage I: 2.8020 (2.0209–3.8849), HR for stage II: 2.9064 (2.3169–3.6459), HR for stage III: 1.6226 (1.2966–2.0307), and HR for stage IV and V: 0.8150 (0.6054–1.0972)). Additionally, our analysis revealed that the effect of drug treatment on primary tumors, recurrent tumors, and metastatic tumors was also different (Supplementary Figure S1). Importantly, once recurrence and metastasis occur, the efficacy of drug treatments becomes less apparent. These findings underscore the importance of timely detection and management of recurrent and metastatic tumors for improving cancer treatment strategies and patient outcomes.

### 3.2 Difference in clinical characteristics of recurrence and metastasis in different stage cancer patients

To further explore the differences in clinical characteristics among early recurrence, early metastasis, and other stages, we investigated their differences in a patient cohort of 8,324 individuals. The cohort was stratified into nine different groups based on the stage and state of cancer progression, including primary tumors, recurrence at stage I, metastasis at stage I, recurrence at stage II, metastasis at stage II, recurrence at stage III, metastasis at stage III, recurrence at stage IV and V, and metastasis at stage IV and V.

**FIGURE 3:**
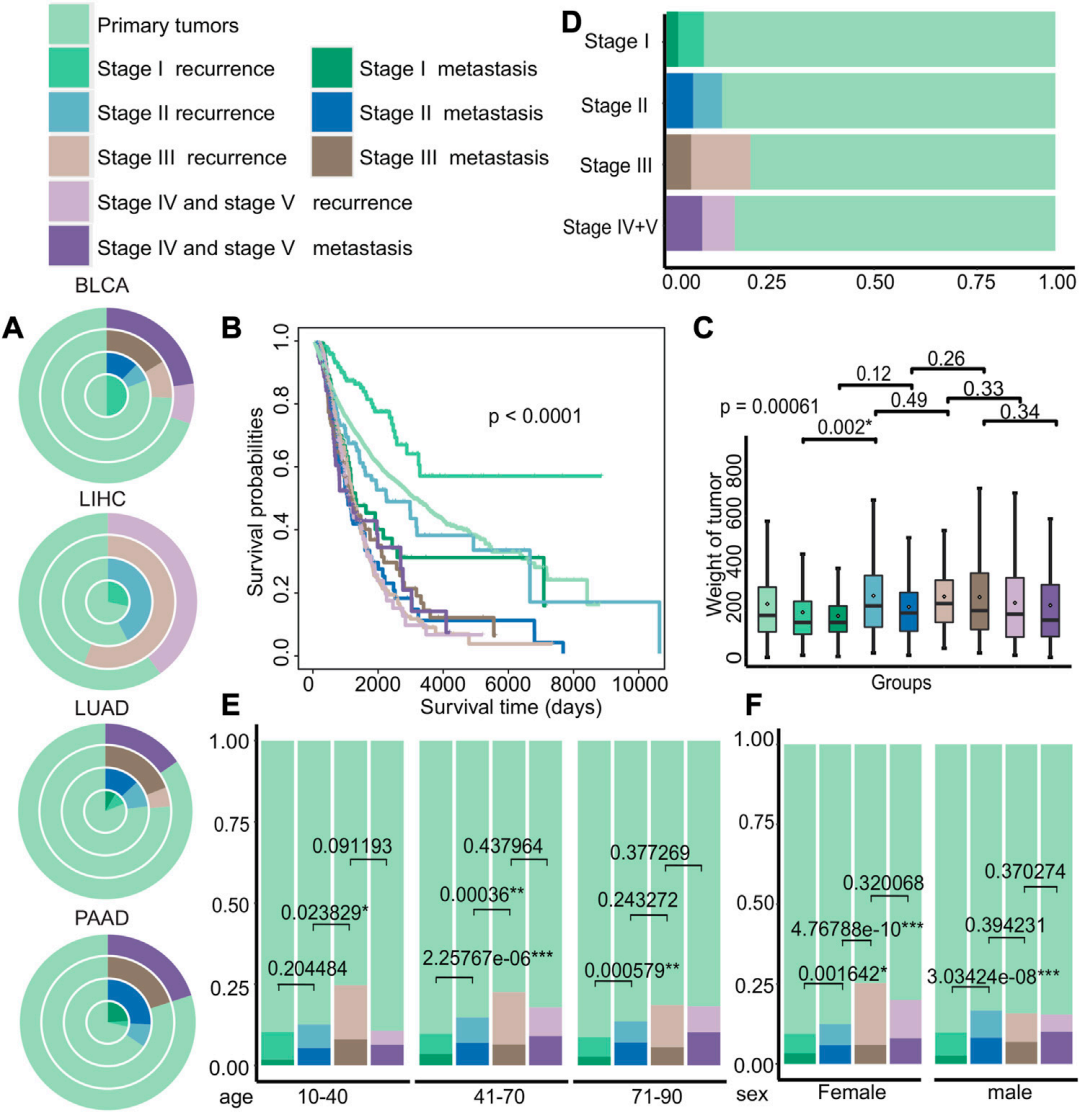
Differences in the clinical characteristics of cancer patients with primary tumors, recurrent tumors, and metastatic tumors. **(A)** The distribution of cancer patients with primary tumors, recurrent tumors, and metastatic tumors in BLCA, LIHC, LUAD, and PAAD in each stage. Each circle from the inside to the outside were stages I, II, III, and IV + V. **(B–C)** Kaplan–Meier survival curves and tumor weight. **(G)** Boxplot of patients with primary tumors, recurrent tumors, and metastatic tumors in various stages. **(D–F)** Distribution histograms on age and gender of patients with primary tumors, recurrent tumors, and metastatic tumors on various stages of cancer. The age groups were divided into young adults aged 10 to 40, middle-aged adults aged 41 to 70, and older adults aged 71 to 90.

Then, we examined the distribution of recurrence and metastasis across different cancer types to identify differences in the incidence of these events in various cancers. Our analysis of TCGA data revealed the difference of recurrence and metastasis in different cancer stages (Figure 3A and Supplementary Figure S2). For example, certain cancers, such as bladder urothelial carcinoma (BLCA) and liver hepatocellular carcinoma (LIHC), had no recorded cases of metastasis patients in certain stages, while lung adenocarcinoma (LUAD) and pancreatic adenocarcinoma (PAAD) had significantly higher rates of metastasis. We also investigated the differences in survival rates among different groups and discovered that the survival of patients with primary tumors, recurrence in stage I, and recurrence in stage II was better, as shown in Figure 3B (*p* < 0.0001). We also observed significant differences in tumor weight between recurrent tumors in stage I and stage II (*p* = 0.002), compared to other stages, with recurrent tumors generally weighing more than metastatic tumors at each stage (Figure 3C). Next, we compared the distribution of groups at different stages of cancer and discovered that the probability of recurrence and metastasis increases as cancer progresses, with the exception of stage IV and stage V (Figure 3D). Furthermore, we analyzed the differences of age and gender among different groups (Figures 3E,F) and discovered that the proportion of stage III recurrence and metastasis was the largest in patients of different age groups, and the proportion of metastases in the remaining stages increased with cancer progression. Meanwhile, the proportion of female patients with stage III recurrence was higher, and the proportion of recurrence and metastasis from stage I to stage II varied significantly regardless of gender (female: *p* = 0.0016 and male: *p* = 3.03*e^−08^).

Our findings suggest that several factors, including age, gender, and type of cancer, may influence the status of recurrence and metastasis of patients with different stages of cancer.

### 3.3 Increased expression of specific genes leads to early recurrence

**FIGURE 4:**
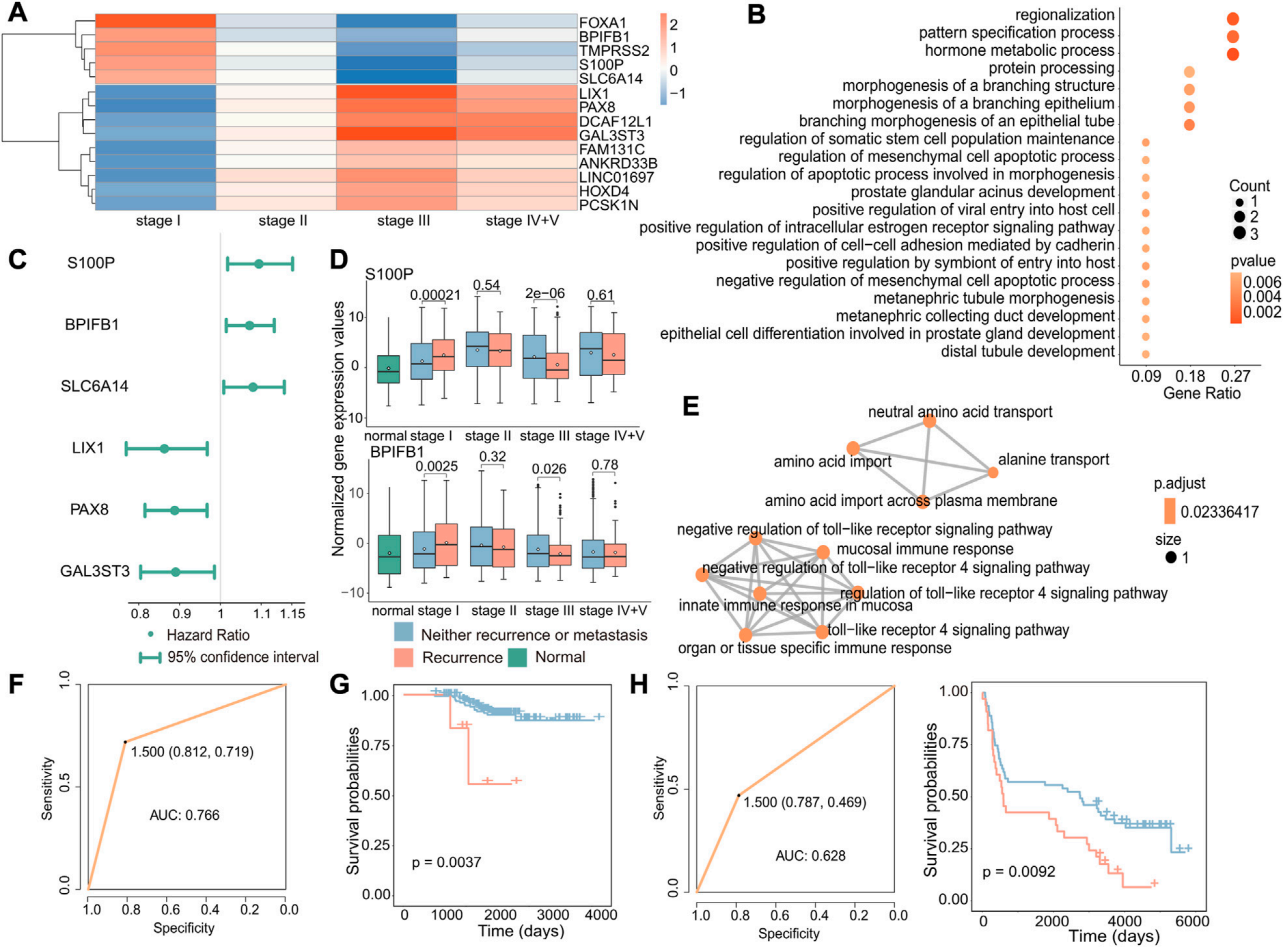
Biomarkers associated with early recurrence in cancer. **(A)** Heatmap of fold-change values of stage-specific differentially expressed genes in stage I. Red indicates that gene expression was upregulated in recurrent tumors, while blue indicates downregulation in recurrent tumors. **(B)** GO enrichment analysis of stage-specific differentially expressed genes in stage I. The size of the dot indicates the number of genes involved in the pathway, and the color of the dot indicates the *p*-value. **(C)** Cox regression analysis of stage I-specific differentially expressed genes related to prognosis. The point is the value of the hazard ratio, and a value greater than 1 indicates that it was related to poor prognosis. **(D)** Boxplot of the expression of S100P and BPIFB1 in normal, primary, and recurrent tumor samples. **(E)** GO enrichment analysis of three genes. Each dot represented a pathway, and the connecting two points meant that the same genes were in the pathways. **(F)** Based on these three genes, the ROC curve of the classifier constructed by the support vector machine method. **(G)** For the GSE31210 dataset, the Kaplan–Meier survival curve of the SVM classification result. Red indicated samples predicted to have recurrence, and blue indicated samples predicted to have no recurrence. **(H)** For the GSE37745 dataset, the ROC curve of the k-nearest neighbor classifier (left) and the survival curve of the classification result (right). Red indicates samples predicted to have recurrence, and blue indicates samples predicted to have no recurrence.

To investigate the potential biomarkers and molecular mechanisms of early recurrence in cancer, we conducted differential expression analysis of gene expression data from primary and recurrent tumors at different stages and identified 14 genes that were specifically differentially expressed in stage I (|log_2_FC|>1, *p* < 0.05, Figure 4A). Next, we performed functional enrichment analysis on these 14 differentially expressed genes using the clusterProfiler package, and enrichment analysis revealed that these genes were associated with biological processes including protein processing and hormone metabolism (Figure 4B). Then, we performed Cox regression analysis to identify genes associated with poor prognosis in recurrence to further test whether these genes can be used as biomarkers to predict recurrence in early cancer (Figure 4C). According to the results of differential expression analysis and Cox regression analysis, we identified three upregulated genes specifically in stage I (*p* = 0.00021, *p* = 0.0025, and *p* = 0.0059), which are associated with poor prognosis in early recurrence (Figure 4D and Supplementary Figure S3). The enrichment analysis results of these three genes (*S100P, BPIFB1*, and *SLC6A14*) showed that the enrichment pathways were roughly divided into two parts, one was transport-related pathways, including BPIFB1 and SLC6A14, and the other was immune-related pathways only including BPIFB1. These associated pathways may contribute to the occurrence of recurrence.

According to previous results, these three genes were not only specific differentially expressed in stage I but also associated with poor prognosis. We further validated the potential of the three identified genes, *S100P, BPIFB1*, and *SLC6A14*, as biomarkers for predicting the recurrence of early-stage cancer. Therefore, we used an SVM to construct the classifier with these three gene expression levels and found the classifier to be effective in predicting the recurrence of early-stage cancer (AUC: 0.766) (Figure 4F). Furthermore, to further verify that these three genes have a general ability to predict the risk of developing early recurrence, we validated the performance of these genes using independent datasets from GEO (GSE31210 and GSE37745) and observed that they exhibited good predictive ability (GSE31210: *p*-value of the difference in survival between the predicted primary and predicted recurrence samples: 0.0037; GSE37745: *p*-value of the difference in survival between the predicted primary and predicted recurrence samples: 0.0092 and AUC: 0.628) (Figures 4G,H).

These findings suggest that *S100P, BPIFB1*, and *SLC6A14* may serve as reliable biomarkers for predicting the risk of recurrence in early-stage cancer. Their overexpression may increase the likelihood of early recurrence.

### 3.4 Identification of diagnostic biomarkers associated with early metastasis

**FIGURE 5:**
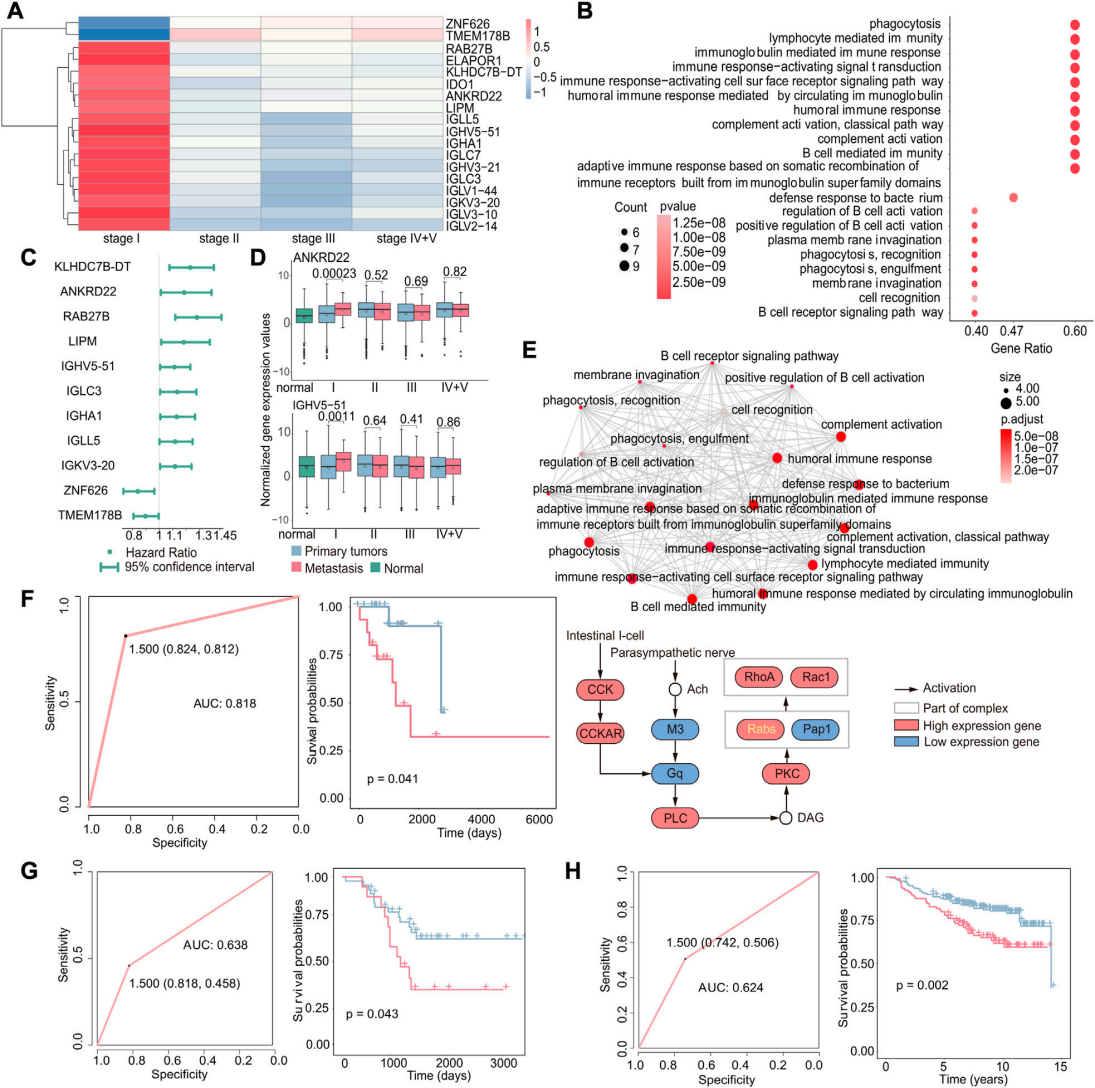
Gene expression-based biomarkers related to early metastasis. **(A)** Heatmap of specific differentially expressed genes in stage I. Red indicates that gene expression was upregulated in metastatic tumors, while blue indicates downregulation. **(B)** GO enrichment analysis of specific differentially expressed genes in stage I. The size of the dot indicates the number of genes involved in the pathway, and the color of the dot indicates the *p*-value. **(C)** Cox regression analysis of stage I-specific differentially expressed genes related to prognosis. The point is the value of the hazard ratio, and a value greater than 1 indicates that it was related to poor prognosis. **(D)** Boxplot of the expression of ANKRD22 and IGHV5-51 in normal, primary, and metastatic tumor samples. **(E)** GO enrichment analysis of nine genes that were related to poor prognosis and specifically differentially expressed in stage I (up). Each dot represents a pathway, and the connecting two points mean that the same genes were in the pathways; KEGG enrichment analysis of the nine genes (down). Specific genes in the KEGG pathway were marked in yellow. **(F)** Based on these nine genes, the ROC curve of the classifier constructed by the support vector machine method and the Kaplan–Meier survival curve of the result of the classifier. Red indicates samples predicted to develop metastasis, and blue indicates samples predicted to have no metastasis. **(G)** For the GSE44295 dataset, the ROC curve of the k-nearest neighbor classifier and the Kaplan–Meier survival curve of the classification result. Red indicates samples predicted to develop metastasis, and blue indicates samples predicted to have no metastasis. **(H)** For the GSE20685 dataset, the ROC curve of the k-nearest neighbor classifier and the Kaplan–Meier survival curve of the classification result. Red indicates samples predicted to develop metastasis and blue indicated samples predicted to have no metastasis.

Identifying differentially expressed genes associated with early-stage metastasis can provide valuable insights into the biological processes driving the metastasis. Therefore, we investigated the genes associated with stage I metastasis using a method similar to that used for studying early cancer recurrence and identified 18 genes that were specifically differentially expressed in stage I (|log_2_FC|>1,*p* < 0.05, Figure 5A); that is, the expression of these genes was only altered in stage I metastatic tumor samples compared with primary tumors, and there were no significant differences in metastatic samples from other stages. Also, it showed the results of enrichment analysis of these 18 genes and found that these pathways were mostly related to immune function (Figure 5B). Interestingly, these pathways were distinct from those identified in the case of recurrence, and it could be attributed to the fact that recurrence involves the emergence of a new tumor in the original site, where the tumor microenvironment is already in balance with the immune system and does not require immune suppression. In contrast, metastasis involves the emergence of a new tumor at a new location, which requires immune suppression to establish and grow. These results suggest that immune-related pathways may play an important role in the development and progression of metastasis.

We further investigated the potential use of these genes for predicting early metastasis in cancer. We used the same methods as the recurrence; according to the results of differential expression analysis and Cox regression analysis (Figure 5C), we identified nine upregulated genes specifically in stage I, including KLHDC78-DT, ANKRD22, RAB27B, LIPM, IGHV5-51, IGLC3, IGHA1, IGLL5, and IGKV3-20 (Figure 5D and Supplementary Figure S3, *p* < 0.05), which are associated with poor prognosis in early metastasis. Gene enrichment results obtained by an emapplot function showed that the nine genes enriched pathways shared many of the same genes and most of them were associated with immune functions that may promote metastasis. Moreover, we identified an RAB27B-related pathway by an enrichKEGG function, which is involved in the transport of intracellular vesicles and proven to be associated with the metastasis (Koh and Song, 2019; Wu et al., 2019).

Above all, these nine genes were not only significantly differentially expressed in stage I cancer but also associated with poor prognosis. Therefore, we constructed a classifier to investigate if these genes could be serve as potential to predict metastasis in early-stage cancer and obtain a good feedback (AUC: 0.818, *p*-value of the survival difference between the predicted primary sample and the predicted metastatic sample: 0.041) (Figure 5F). In order to verify the accuracy of the classifier, the gene expression data and relevant clinical data of GSE44295 and GSE20685 were downloaded from the GEO database for verification (GSE44295: *p*-value of the difference in survival between the predicted primary and predicted metastatic samples: 0.043 and AUC: 0.638; GSE20685: *p*-value of the difference in survival between the predicted primary and predicted metastatic samples: 0.002 and AUC: 0.624) (Figures 5G,H). The results demonstrated that the classifier was effective in predicting metastasis, which suggests that these genes could be potential targets for developing diagnostic strategies for early cancer metastasis.

### 3.5 Identification of a gene mutation signature for early metastasis prediction

**FIGURE 6:**
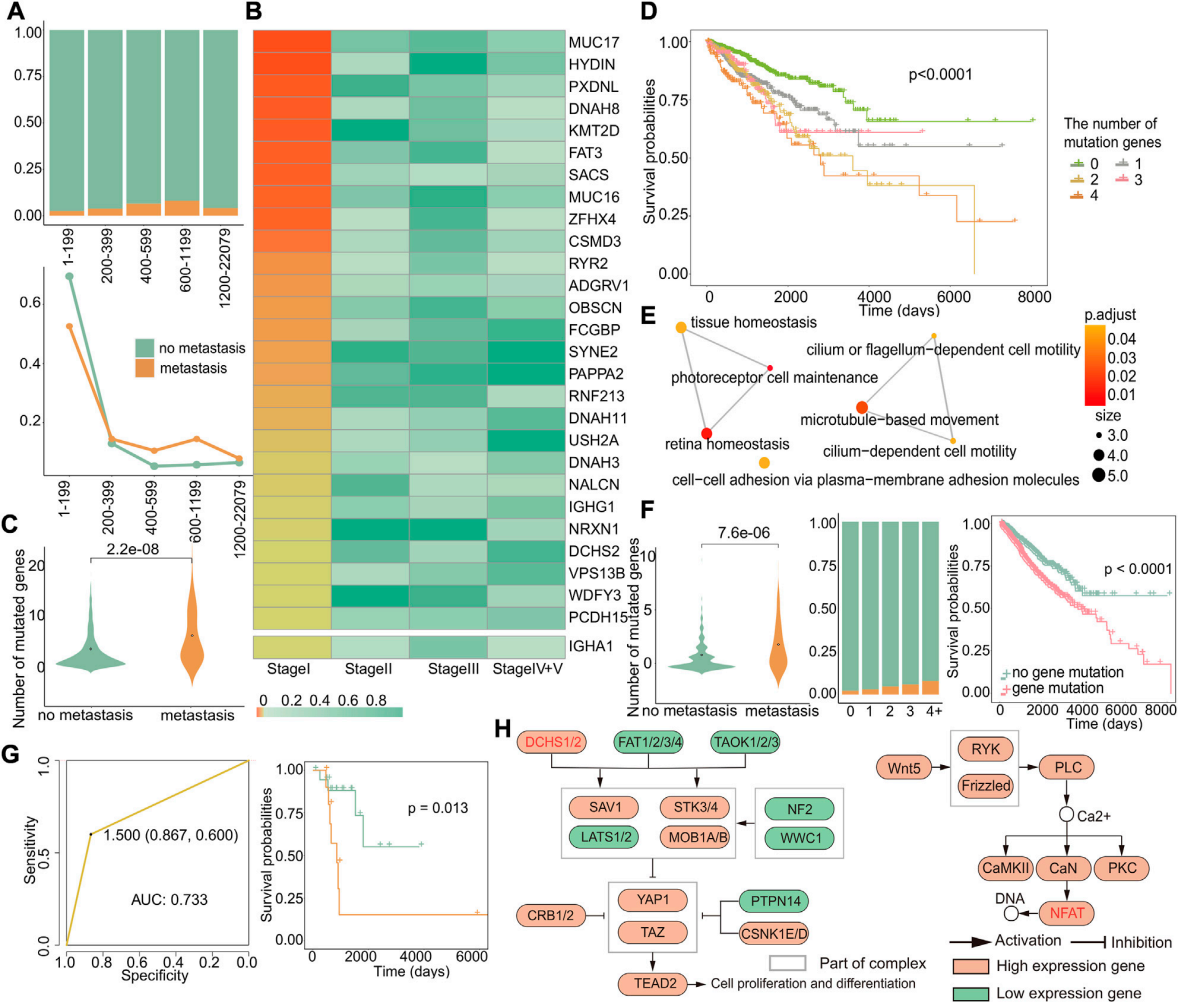
Genes specifically mutated in early metastasis. **(A)** The distribution of primary and metastatic tumors in different mutation number groups (up). Changes in the distribution of primary and metastatic tumors in the total primary and metastatic tumors in different mutation groups (down). The abscissa represented groups with different numbers of mutant genes. **(B)** Heatmap of Fisher’s exact test *p*-value of mutant genes related to specific metastasis in stage I. **(C)** For these 28 genes, the difference in the number of mutations between the primary and metastatic tumor. **(D)** Kaplan–Meier survival curves of samples with different numbers of mutations. **(E)** GO enrichment analysis of 28 genes. Each dot represents a pathway, and the connecting two points mean that the same genes were in the pathways. **(F)** For these 12 genes, left: difference in number of mutations between primary and metastatic tumor samples; middle: the proportion of primary and metastatic tumors in groups with a different number of mutations, where green was the primary tumor and yellow was the metastatic tumor; and right: survival differences in samples with or without genetic mutations. **(G)** Based on 12 genes, the ROC curve of the classifier constructed by k-nearest neighbors and the Kaplan–Meier survival curve of the result of the classifier. Yellow indicates samples predicted to develop metastasis, and blue indicates samples predicted to have no metastasis. **(H)** KEGG enrichment analysis of 12 genes. Related genes are marked in red.

To gain deeper insights into the molecular mechanisms underlying early recurrence and early metastasis, we performed additional analyses to identify a specific signature from the angle of gene mutations that could potentially serve as biomarkers for early recurrence and early metastasis. First, we investigated the relationship between the number of gene mutations and the likelihood of early metastasis by studying the ratio of metastasis to primary in different mutation groups. The results revealed that tumors with a number of gene mutations within the range of 600–1,199 were most prone to metastasis, as demonstrated by the highest proportion of metastasis in this group (Figure 6A). To further verify this result, we respectively calculated the proportion of metastasis or primary in the total metastasis or total primary in each group and found that except for the first group, the proportion of metastatic samples in 600–1,199 was indeed the largest (Figure 6A). Although groups 1–199 had the largest proportion of metastatic samples, this was due to an imbalance caused by the large number of samples in this group, and the proportion of metastatic tumors in this group was very small and, thus, could be ignored. Our results suggest that tumors with 600–1,199 gene mutations are most likely to metastasize.

Then, we used Fisher’s exact test to identify specific mutation genes (Section 2.13) that exhibited a significant difference only in stage I, with a *p*-value less than 0.05 only in stage I and greater than 0.05 in the other stages. We did not observe an overlap between genes with specific mutations and high-frequency mutation genes with a mutation rate greater than 5% in cases of early recurrence. However, in metastasis, we identified 27 genes with both specific mutations and high-frequency mutations in stage I (Figure 6B). Furthermore, when combined with significantly differentially expressed genes in stage I metastasis, we discovered that IGHA1 was a gene that had mutations and expression specifically in stage I, even though it was not a high-frequency mutation gene. Among these 28 genes, there were more early metastasis mutations than primary tumors (*p* = 2.2*e^−08^), which could cause worse prognosis (Figure 6C). In order to verify whether the higher the number of mutations, the worse the prognosis, we grouped the primary and metastatic samples according to the number of mutations in these 28 genes and analyzed the survival difference between the groups and found that the number of mutated genes was positively correlated with the worse prognosis (*p* < 0.0001) (Figure 6D). Next, the result of Go enrichment analysis suggested that the 12 genes, namely, *FAT3, NRXN1, DCHS2, PCDH15, HYDIN, DNAH8, SYNE2, DNAH11, DNAH3, ADGRV1, USH2A*, and *IGHA1*, were mainly involved in homeostasis, cell motility, and cell adhesion pathways (Figure 6E), which are known to play a critical role in metastasis (Zhou and Huang, 2011; Bhatia et al., 2019; Läubli and Borsig, 2019). Interestingly, the analysis of these 12 genes showed that there were more gene mutations in early metastatic patients (*p* = 7.6*e^−06^), and the proportion of early metastatic samples increased with the increase in the number of mutated genes. Moreover, patients with mutations of these 12 genes showed significant differences in survival from those without mutations (*p* < 0.0001), indicating that these genes can serve as biomarkers for early metastasis (Figure 6F). So, we constructed a classifier based on these 12 genes, and the results of the classifier showed that the mutations in these genes can distinguish patients with or without metastasis, with an AUC of 0.733 and a *p*-value of 0.013 for the survival difference between the predicted primary and the predicted metastatic samples (Figure 6G). Then, we investigated the relationship between these 12 genes and early metastasis in cancer by pathway analysis. Gene enrichment analysis for KEGG revealed two pathways, the Hippo pathway, where DCHS2 was located, and the WNT/calcium pathway, where FAT3 was located, both of which are widely believed to be closely related to cancer (Figure 6H) (Sanchez-Vega et al., 2018).

These findings suggest that mutations in these 12 genes may impact early metastasis and could serve as potential biomarkers for early detection. Importantly, our results also indicate that there is no specific mutation in stage I that could affect recurrence, highlighting the fact that early metastatic tumors exhibit more differences in mutations compared to primary tumors.

### 3.6 Specific copy number variation in the early stage may drive early metastasis in cancer

**FIGURE 7:**
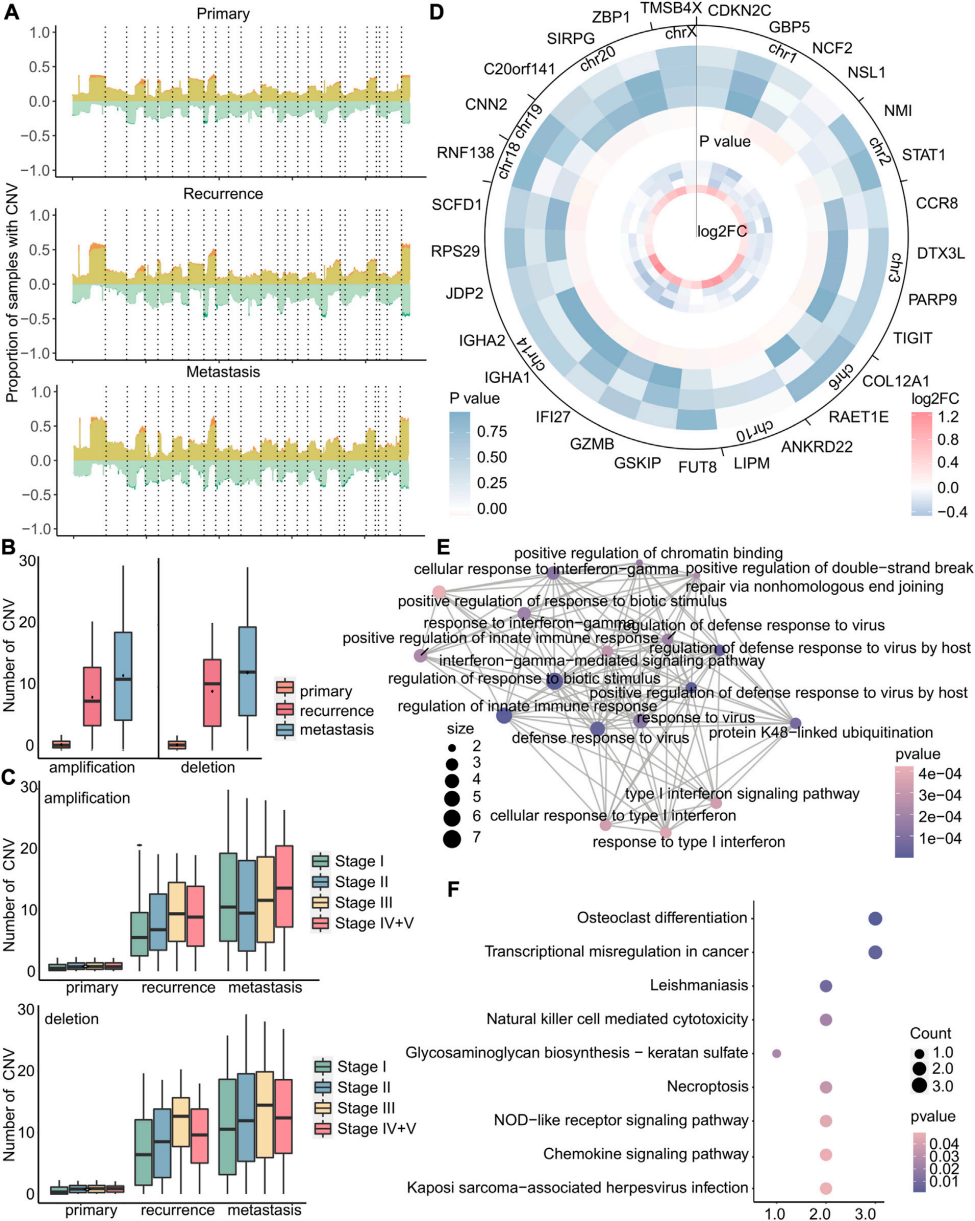
Copy number variation in stage I metastatic tumors is more complicated. **(A)** For primary, recurrent, and metastatic samples of stage I, the proportion of samples with copy number variations to the total samples. The upper part of 0 indicates the copy number amplification rate, which was the sum of the low-level copy number amplification rate in light yellow, and the high-level copy number amplification rate is in dark yellow. The lower part of 0 indicated the copy number deletion rate, which was zero minus its true value. The mutation rate is the sum of the single-copy deletion rate in light green, and the homozygous deletion rate is in dark green. Dotted lines separated different chromosomes. **(B–C)** Boxplot of the number of amplifications or deletions in each sample. **(D)** In metastatic tumors, heatmap of the log_2_FC value of specific copy number variant genes in stage I (small) and heatmap of Fisher’s exact test *p*-value (large). Stages I, II, III, and IV + V of cancer from the inside to the outside. **(E–F)** GO enrichment analysis and KEGG enrichment analysis of 29 genes. Each dot represents a pathway, and the connecting two points mean that the same genes were in the pathways. The size of the dot indicates the number of genes in these 29 genes, and the color of the dot indicates the *p*-value.

CNV is a type of genetic variation that can lead to amplification or deletion of genes and is closely related to cancer (Liang et al., 2016). Therefore, we investigated the differences of copy number variation on all chromosomes at each stage of primary tumor, recurrent tumor, and metastatic tumor and found that the copy number variation rates of specific genes, such as those located in chromosomes 1 and 8, were observed to increase significantly at each stage of recurrent and metastatic tumors compared to primary tumors (Figure 7A and Supplementary Figure S4). Also, the copy number variations of recurrent and metastatic tumors are significantly higher than those of primary tumors, and metastatic tumors have the most copy number variations (Figure 7B). In addition, the patients of stage I had consistently lower numbers of copy number deletions compared to other stages, while the numbers of copy number amplifications in stage I metastasis were relatively higher (Figure 7C), indicating a more intricate environment. Therefore, we then focused on amplification in stage I metastasis and identified 5,858 genes with specific amplification in stage I that may affect metastasis, using Fisher’s exact test (*p* < 0.05). As amplification typically leads to upregulation of gene expression, we further selected 29 genes from these 5,858 genes with upregulated expression in stage I (|log_2_FC| > 0.8) and downregulated expression in other stages (Figure 7D). The enrichment results of these 29 genes revealed significant associations with immune-related and anti-viral functions, as demonstrated by the GO analysis results (Figure 7E), Additionally, it highlighted the involvement of these genes in various cell-mediated functions and transcriptional disorders as demonstrated by KEGG analysis results (Figure 7F).

In brief, primary tumors, recurrent tumors, and metastatic tumors exhibit significant differences in copy number variation. Amplification of stage I-specific genes may lead to early metastasis, while there were no specific copy number variations associated with early recurrence. Our study also revealed that the genome of early recurrence may not differ significantly from that of the primary tumor in terms of mutation and copy number variation. Therefore, the high variability of the genome may contribute to the occurrence of early metastasis, emphasizing the need for identifying biomarkers associated with early metastasis.

### 3.7 Identification of tumor microenvironment characteristics associated with early recurrence and early metastasis

**FIGURE 8:**
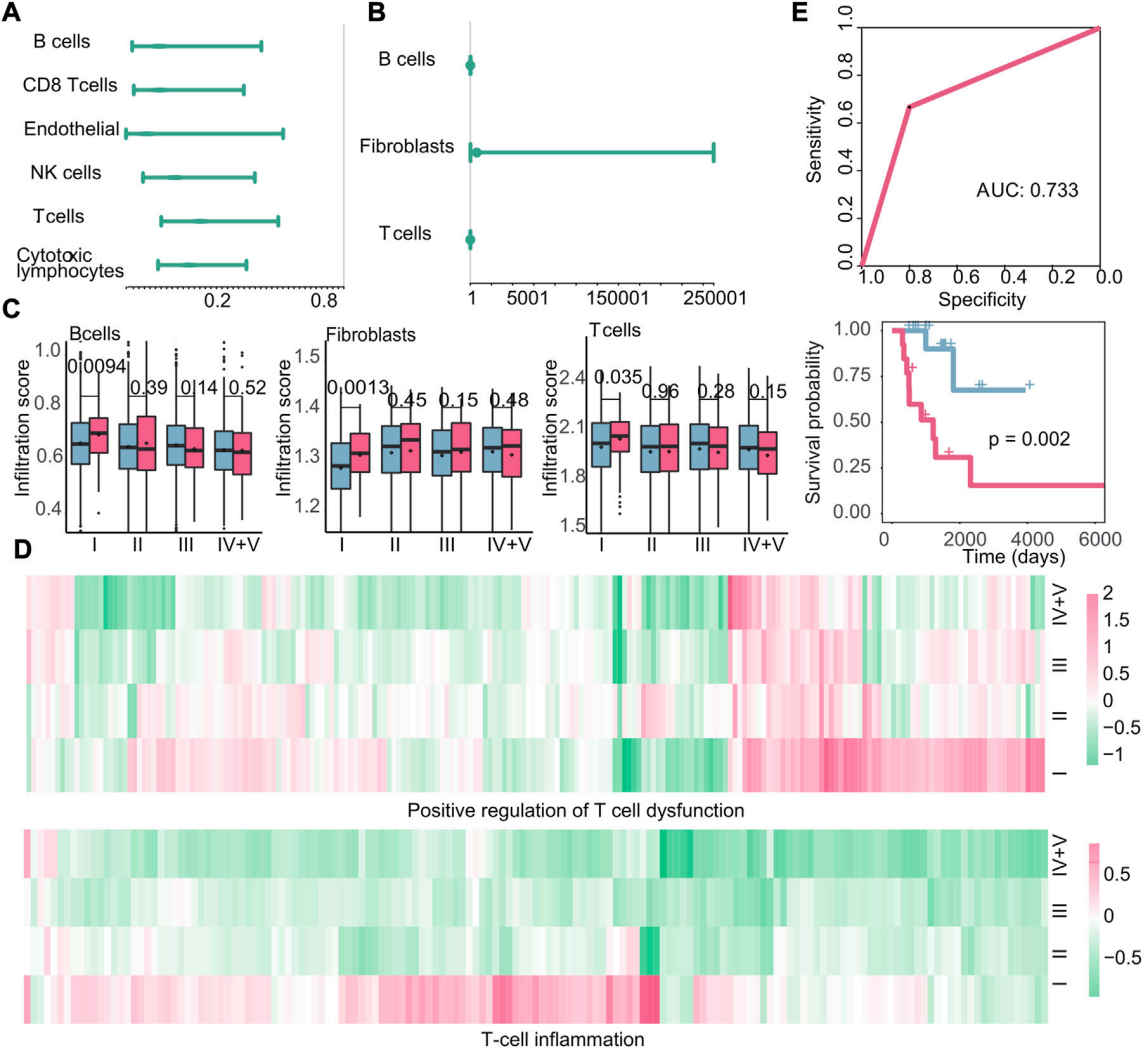
Immune microenvironment and immune characteristics. **(A–B)** Cox regression analysis of immune cell infiltration in recurrent tumors or metastatic tumors. At this time, the endpoint event was the generation of new tumors. The dot represents the hazard ratio, and having a value greater than 1 represents the promotion of new tumors. **(C)**: Boxplots of the infiltration of B cells, fibroblasts, and T cells in primary and metastatic tumors at various stages. Blue is the primary tumor, and red is the metastatic tumor. **(D)** Heatmap of log2FC values of genes related to positive regulation of T-cell dysfunction and genes related to T-cell inflammation calculated based on gene expression data. **(E)** Based on the infiltration of the three types of cells, the ROC curve of the classifier constructed by k-nearest neighbors and the Kaplan–Meier survival curve of the result of the classifier. Red indicates samples predicted to develop metastasis, and blue indicates samples predicted to have no metastasis.

The investigation of the tumor microenvironment and immune status in relation to early recurrence and early metastasis is crucial for gaining new insights into cancer progression. To investigate the impact of immune cell infiltration on early recurrence and early metastasis, we calculated the cell infiltration score of 13 cell types (Section 2.5). Based on the immune cell infiltration scores, we used Cox regression analysis to study the relationship between immune cells and new tumor generation and found that a negative correlation between six immune cell infiltration scores and the production of new tumors in stage I recurrence (Figure 8A). However, we did not detect any immune cell types that exhibited a specific association with the recurrence of stage I tumors; in other words, the infiltration of these six immune cells may affect the recurrence of cancer at all stages, not just in stage I. Meanwhile, three kinds of immune cell (B cells, fibroblasts, and T cells) were observed as cells that promote the production of new tumors in stage I metastasis by Cox regression analysis (Figure 8B). Also, we compared the difference of cell infiltration level between different cancer stages with the *t*-test and discovered that B cells, fibroblasts, and T cells in stage I metastasis present specific significant high cell infiltration, which suggest that the high levels of three cells infiltrating may promote early metastasis (*p* = 0.0094, *p* = 0.0013, and *p* = 0.035) (Figure 8C). Notably, according to our findings, high infiltration of immune cells may lead to metastasis instead, and for this, we further investigated the expression changes of T-cell dysfunction and T-cell inflammatory genes in metastasis patients at different stages, in comparison to the primary tumor. The results demonstrated that the genes related to T-cell inflammation and positive regulation of T-cell dysfunction in these cells were specifically upregulated in stage I metastasis (Figure 8D). This finding suggest that despite the high enrichment of T cell in the tumor microenvironment of patients with metastasis in early tumor, most infiltrating T cells underwent functional dysfunction and inflammation, which indicated that T cells were likely to be assimilated by cancer cells during this stage, thereby jointly promoting poor prognosis. Subsequently, a classifier was constructed using the k-nearest neighbor method based on the aforementioned immune cells (AUC: 0.733, *p*-value of the survival difference between the predicted primary sample and the predicted metastatic sample: 0.002) (Figure 8E), suggesting that the infiltration of these three cells could potentially impact early metastasis.

**FIGURE 9:**
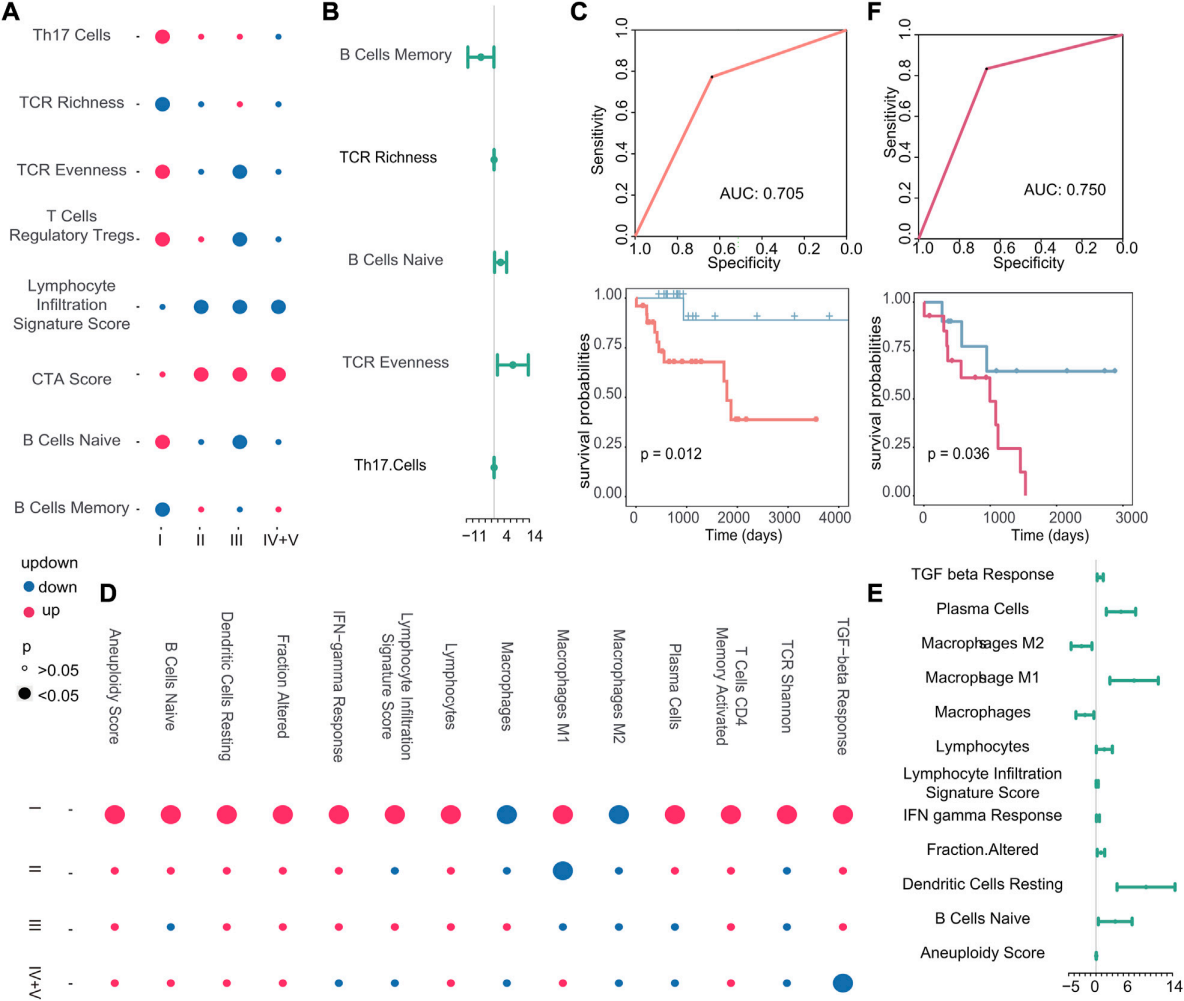
**(A–D)** Dot plots of stage I-specific immune characteristics in recurrent and metastatic tumors. The color of the dot indicates the change in the score of the immune characteristics, and the size of the dot indicates the *p*-value. **(B–E)** Cox regression analysis of stage I-specific immune characteristics in recurrent and metastatic tumors. The endpoint event at this time was the generation of new tumors. The dot represents the logarithmic transformation of the HR value, and greater than 0 indicates that it promoted the generation of new tumors. **(C–F)** Based on the immune characteristics that promote the generation of new tumors, the ROC curve of the classifier constructed by k-nearest neighbors and the Kaplan–Meier survival curve of the result of the classifier. Red indicates samples predicted to develop metastasis, and blue indicates samples predicted to have no metastasis.

Next, we examined the correlation between other immune characteristics and the occurrence of early recurrence and early metastasis in stage I tumors. According to the scores of immune characteristics, we calculated the difference *p*-value and up–downregulation between patients with recurrence or metastasis, and primary patients at each cancer stage through difference analysis, we identified specific immune characteristics that were positively associated with the emergence of new tumors in stage I, as shown in Figure 9A. In particular, three immune characteristics (TCR richness, B cells naive, and TCR evenness) related to the emergence of new tumors were identified as biomarkers associated with early recurrence and Cox regression analysis (Figure 9B). Then, the scores of these three features were used to construct a classifier to further verify their good performance as biomarkers (AUC: 0.705, *p*-value of the difference in survival between the predicted primary and predicted recurrence samples: 0.012) (Figure 9C). Similarly, in the case of metastasis, 14 specific immune characteristics in stage I were found through the scores of immune characteristics by difference analysis (Figure 9D). Also, 10 of them were positively associated with the generation of new tumors in stage I, which are considered to be possible biomarkers of early metastasis (Figure 9E). Then, the classifier we constructed confirmed the good performance of these immune characteristics in predicting early metastasis (AUC: 0.750, *p*-value of the difference in survival between the predicted primary and predicted metastasis samples: 0.036) (Figure 9F).

In summary, we found that the high infiltration of B cells, T cells, and fibroblasts is positively correlated with early metastasis, while no immune cells may serve as a potential biomarker for early recurrence. Furthermore, we identified specific immune characteristics unique to stage I tumors, such as B-cell naive proliferation, which appeared to contribute to an increased risk of early recurrence, and macrophage M1 polarization, which appeared to contribute to an increased risk of early metastasis. Our findings present the potential of immune characteristics as useful biomarkers for predicting the likelihood of tumor recurrence and metastasis in stage I cancers.

### 3.8 Primary, recurrent, and metastatic tumors exhibit distinct cellular states

**FIGURE 10:**
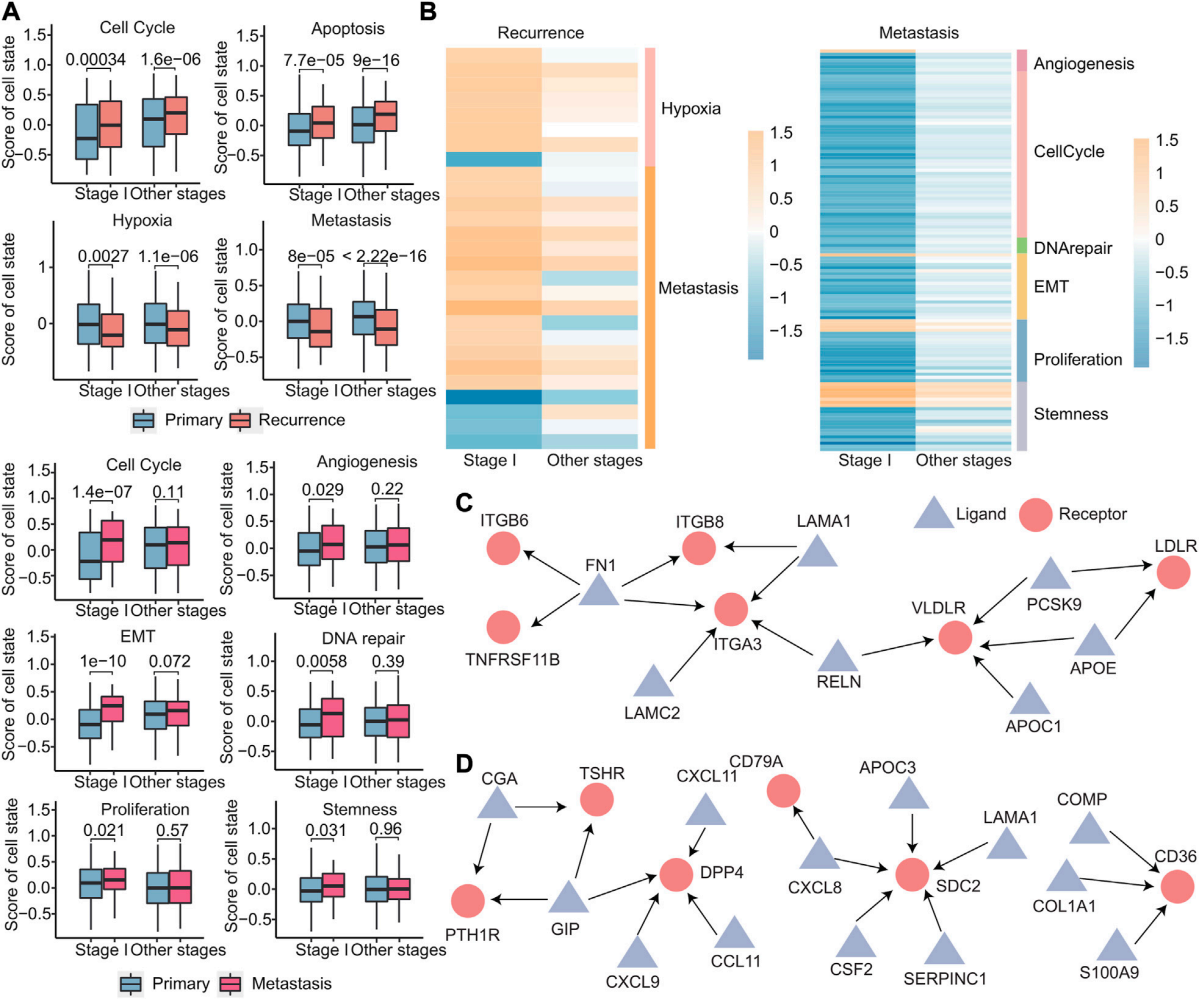
Differences in primary, recurrent, and metastatic tumors at the cellular level. **(A)** Boxplot of the scores calculated by the cell state-related gene set for each sample. **(B)** Heatmap of log_2_FC values of genes related to cell status calculated based on gene expression data. The cell status type was indicated on the right. **(C–D)** Specific ligand–receptor pair networks for stage I recurrence **(C)** and metastasis **(D)**. The triangular points are the ligands, and the round points are the receptors.

The cellular states of primary, recurrent, and metastatic tumors play a critical role in cancer progression and treatment and in order to gain insights into the cellular states that are involved in early recurrence and early metastasis of cancer; we identified different cell states in early recurrence and early metastasis. Using the expression of 14 cell state-related genes, we calculated the cell state scores for each patient using GSVA. We identified four cell states (cell cycle, apoptosis, hypoxia, and metastasis) that were different in stage I and other stages of recurrence than in the primary tumor, that is, there was no stage I recurrence-specific cell state. Meanwhile, six cell states (cell cycle, angiogenesis, epithelial–mesenchymal transition, DNA repair, proliferation, and stemness) were significantly different in stage I metastasis (Figure 10A). Notably, these six cell states were not significantly different in other stages, indicating that cell states were more specific in early metastasis than in early recurrence. Furthermore, these four cell states in early recurrence were primarily related to the microenvironment, while these six cell states in early metastasis were primarily related to the tumor’s invasiveness, which more clearly explained the two different states of early recurrence and early metastasis. To further investigate these cell states, we analyzed the signature genes associated with each state by differential expression analysis and discovered that 27 genes specific for early recurrence and 128 genes specific for early metastasis that were differentially expressed in stage I when compared with other stages (Figure 10B and Supplementary Figure S5).

Ligand–receptor interactions between cells can help us understand the heterogeneity of tumor microenvironment better, so the 3,398 ligand–receptor pairs were then analyzed. We first identified stage I-specific ligand–receptor pairs in early recurrence and early metastasis. These genes exhibited differential expressed in stage I (|log_2_FC| > 0.8 and *p* < 0.05), with no expression differences in other stages. Then, for the networks comprising these ligand–receptor pairs, we further screened out hub nodes in networks, which had a degree no less than three (hub nodes of recurrence: FN1, ITGA3, and VLDLR and hub nodes of metastasis: GIP, DPP4, SDC2, and CD36) (Figures 10C,D). These hub nodes were all confirmed to be associated with recurrence (Gong et al., 2018; Zhai et al., 2019) and metastasis (Anderluh et al., 2016; Pascual et al., 2017; Tsoyi et al., 2019; Du et al., 2020; Hua et al., 2020).

These results demonstrate that early metastasis exhibits greater divergence from the primary tumor in terms of changes in cellular states compared to early recurrence. Moreover, the analysis of ligand–receptor interactions between cells can help better understand the heterogeneity of the tumor microenvironment. Targeting these hub nodes could potentially inhibit early recurrence and early metastasis by affecting adjacent genes in the network.

### 3.9 Targeted therapy and immune checkpoint inhibitors show promise for improved responses in patients with early metastasis

**FIGURE 11:**
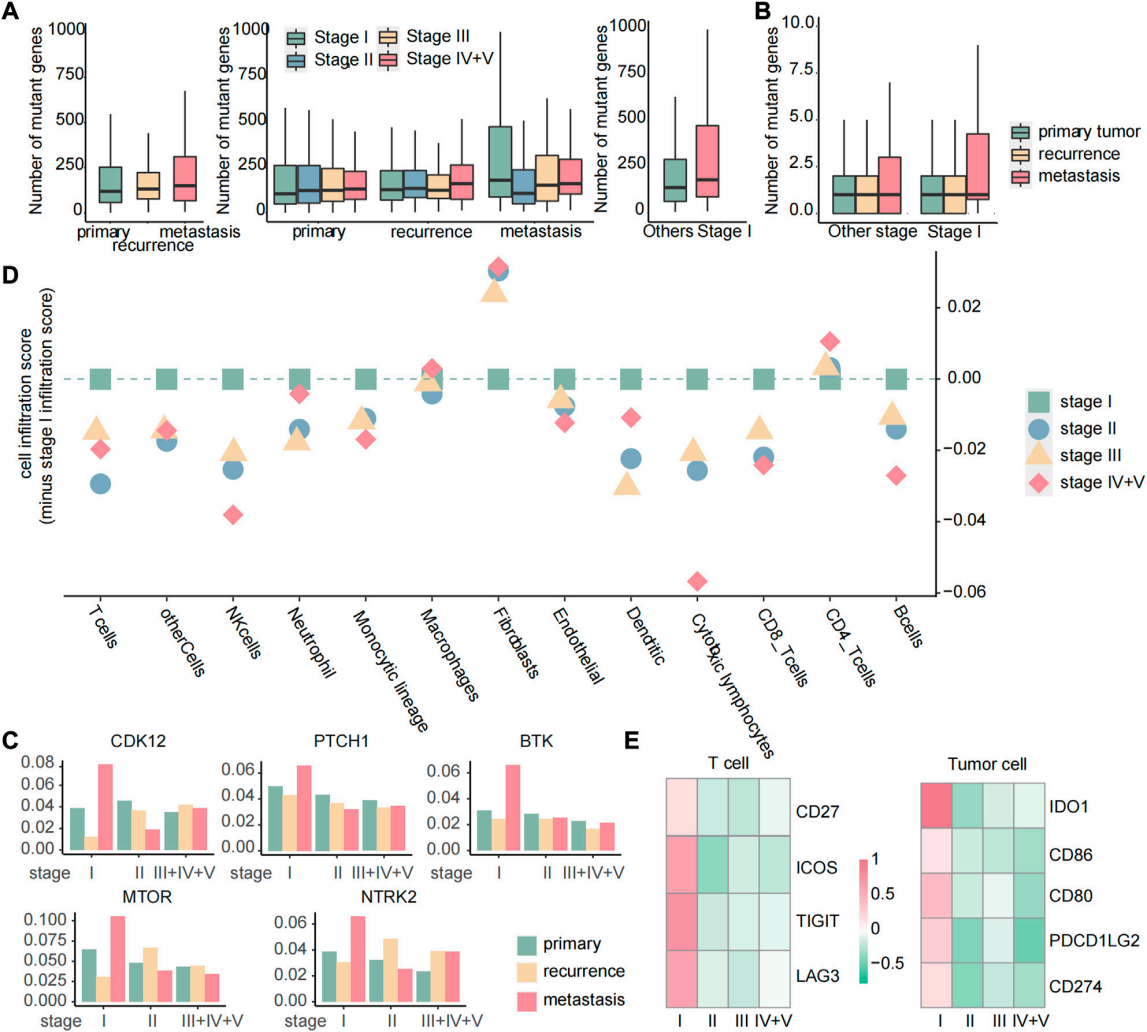
Targeted therapy and immune checkpoint therapy are more effective for early metastases. **(A)** Boxplot of the number of mutated genes in primary, recurrent, and metastatic tumors, boxplot of the number of mutated genes in primary, recurrent, and metastatic tumors of each stage, boxplot of the number of mutated genes in metastatic tumors of stage I and other stages. **(B)** For 158 genes related to targeted therapy, boxplot of the number of mutated genes in each sample. **(C)** Histogram of the mutation rate of specific targeted therapy-sensitive genes. The samples were divided into primary, recurrent, and metastatic samples at different stages. **(D)** Dot plot of the score of immune cell infiltration in metastatic tumors. All the scores were reduced by the cell infiltration fraction of stage I. If the scores of other stages were less than 0, we considered that the cell had the highest infiltration level in stage I metastatic tumor. **(E)** Heatmap of FC values of genes related to immune checkpoint calculated based on gene expression data.

Targeted therapy is a kind of treatment at the molecular level and can be tailored to each patient’s specific genetic mutations, making it a personalized approach that can potentially improve patients’ quality of life and survival rates (Lee et al., 2018). To identify the potential response of targeted therapy, we analyzed the number of gene mutations in primary, recurrent, and metastatic tumors. We discovered that the number of mutated genes exhibited different patterns of increase across primary, recurrent, and metastatic tumors. Specifically, stage I metastatic tumors showed a significant increase in the number of mutated genes, while primary tumors displayed a gradual increase with tumor development. In recurrent tumors, the number of mutated genes increased with tumor development, except in stage III tumors. Subsequently, we further compared the number of mutated genes in stage I metastatic tumors with those in other stages and found that the number of mutated genes in stage I metastatic tumors was significantly higher than those in other stages (Figure 11A). These results suggest that stage I metastatic tumors may respond better to targeted therapy. Among the 158 genes associated with targeted therapy obtained from the OncoKB database, there were still the most mutated genes in stage I metastatic tumors (Figure 11B). Moreover, by calculating the mutation rate of genes, we identified five genes that exhibited specific sensitivity to targeted therapy in stage I metastatic tumors. These five genes have particularly high mutation rates of greater than 5% in stage I metastases, and these genes have therapeutic information (Figure 11C). Suppose that mutations in the twelve biomarker genes observed through gene mutation data were detected in early cancer stages. In this case, targeted therapy with corresponding drugs for the aforementioned five gene loci may be considered to delay or inhibit the occurrence of metastasis and improve prognosis. Specifically, C481S, C481F, C481Y, T316A, T474I, and T474S mutate of BTK could be affected by ibrutinib to improve the prognosis of patients with chronic lymphocytic leukemia or small lymphocytic lymphoma. Olaparib can be used to inhibit oncogenic mutations or truncating mutations of CDK12 to reduce the risk of prostate cancer or NOS metastasis. Sonidegib can improve the prognosis of patients with embryonal tumor by inhibiting PTCH1 truncating mutations. Everolimus or temsirolimus can improve the prognosis of patients with all solid tumors by affecting the mutation status of mTOR. Fusions of NTRK2 were inhibited by entrectinib or larotrectinib to reduce the risk of metastasis in patients with all solid tumors.

Immune checkpoint inhibitor therapy has proven to be one of the most promising and effective immunotherapies in recent years by modulating immune cells to inhibit tumor (He et al., 2021). In order to analyze the difference of cellular infiltration between stage I metastasis and other stages, the 13 immune cell infiltration scores in stage I of the metastatic tumor samples were subtracted from the score of each stage, and we found that the majority of immune cells showed a highly cell infiltrated level at stage I metastasis (Figure 11D), suggesting a favorable response to immune checkpoint therapy in patients at this stage. Genes associated with immune checkpoints obtained from the GeneCards database were then studied through differential expression analysis, and four genes in T cells and five genes in tumor cells were observed to be specifically upregulated in stage I metastasis (|log_2_FC| > 0.8 and *p* < 0.05) (Figure 11E), which are associated with immune checkpoints. Similarly, suppose the three immune cells we identified as biomarkers were detected to be highly infiltrated in early cancers. In this case, personalized immune checkpoint therapy with corresponding drugs for the aforementioned nine gene loci in different patients may be considered to delay or inhibit the occurrence of metastasis and improve prognosis (Supplementary Table S1).

Overall, when patients with high risk of metastasis in stage I tumors are identified, personalized targeted or immune checkpoint therapy with corresponding drugs for the aforementioned specific gene loci may be considered to delay or inhibit the occurrence of metastasis and improve prognosis. It is important to note that the effectiveness of immune checkpoint therapy and targeted therapy may vary depending on the individual patient’s cancer type and status. Therefore, careful consideration of each patient’s unique circumstances is necessary to determine the optimal treatment approach. By providing timely and effective treatment, we can more comprehensively and effectively control disease’s development and improve patient outcomes.

## 4 Discussion

Our study aimed to identify biomarkers related to early recurrence and early metastasis in pan-cancer patients by analyzing multi-omics data. These findings highlight the importance of identifying these biomarkers as they could aid in the detection of cancer patients with a high risk of early recurrence and metastasis. We observed significant differences in the distribution of various factors between patients with and without recurrence or metastasis in early cancer, indicating the importance of identifying high-risk patients for early intervention. We also identified several specific genes related to early recurrence and metastasis that, when overexpressed, could promote their occurrence. Additionally, mutations and copy number amplifications in certain genes were observed to increase the risk of early metastasis. Interestingly, by comparing the genes identified in the genome and the transcriptome, we discovered that there were intersections between these genes. The overexpression and copy number amplification of ANKRD22 and LIPM in early cancer, and overexpression, mutation, and copy number amplification of IGHA1 in early cancer may all promote early metastasis. The reasons for this intersection are worth investigating further. Furthermore, we identified immune characteristics related to early recurrence and early metastasis and also found treatment-sensitive genes that may help improve therapeutic outcomes for cancer patients.

Our findings are consistent with previous studies that have identified specific genes and pathways associated with cancer recurrence and metastasis. For example, a study by Hwang et al. (2021) found that overexpression of the gene *S100P* was associated with an increased risk of early recurrence and poor prognosis in patients with hepatocellular carcinomas . Hendrix et al. demonstrated that the gene *RAB27B* was associated with metastasis of breast cancer (Hendrix et al., 2010). Other studies have identified mutated genes associated with early recurrence, including FAT3 and USH2A (Kim et al., 2021; Qiu et al., 2021). In addition, other researchers have also used multi-omics data to identify biomarkers for cancer recurrence and metastasis. For example, a study by Aftimos et al. (2021) used genomic and transcriptomic data to identify a set of genes associated with metastasis in breast cancer. Another study by Yang et al. (2021) found that changes in copy number and expression of gene and changes in infiltration of immune cells may be associated with early metastasis in pancreatic ductal adenocarcinoma by using multi-omics data.

Our study adds to the growing body of the literature on the use of multi-omics data to identify biomarkers for early cancer recurrence and metastasis. By integrating transcriptomic, genomic, and immune cell data, we were able to identify specific genes and characteristics associated with early recurrence and early metastasis in pan-cancer. These findings may provide insights into the underlying mechanisms of early cancer recurrence and metastasis and may have implications for the development of new strategies for early cancer recurrence and metastasis identification.

One of the strengths of our study is the integration of transcriptomic, genomic, and immune cell data to identify specific genes and characteristics associated with early recurrence and early metastasis in pan-cancer. However, there are some limitations that should be considered. One limitation of our study is the exclusion of several cancer types due to the lack of pathological stage information. Although our study includes most cancer types, it is possible that relevant biomarkers for early recurrence and early metastasis may have been missed. Therefore, future studies should consider obtaining more comprehensive pathological stage information to improve the accuracy of the results. Another limitation of our study is the lack of treatment response data in TCGA. While we identified treatment-sensitive genes, the lack of relevant data may have biased our results. Future studies should aim to incorporate more comprehensive treatment response data to better understand the relationship between treatment and early recurrence and early metastasis. Furthermore, our study only analyzed transcriptomic, genomic, and immune cell data. Additional omics data, such as epigenomics and proteomics, may provide additional insights into the mechanisms of early recurrence and early metastasis in cancer. Therefore, future studies should aim to incorporate other omics data to gain a more comprehensive understanding of these processes.

Despite these limitations, our study adds to the growing body of literature on the use of multi-omics data to identify biomarkers for early cancer recurrence and metastasis. Our findings may have implications for the development of new therapies and strategies for the identification of early cancer recurrence and metastasis. Not only that, future studies should also aim to incorporate other omics data, such as epigenomics and proteomics, to further understand the mechanisms of early recurrence and early metastasis in cancer.

## 5 Conclusion

In summary, our multi-omics analysis reveals that early recurrence and early metastasis in various cancer types have distinct molecular mechanisms and tumor microenvironments. The biomarkers identified through our multi-omics analysis have context-dependent prognostic implications and potential as targets for predicting the risk of early recurrence and early metastasis. These findings provide new insights into the underlying biology of cancer progression and may have implications for the development of personalized therapies for cancer patients.

## Data Availability

The original contributions presented in the study are included in the article/Supplementary Material; further inquiries can be directed to the corresponding authors.

## Supplementary material

Supplementary Figure S1 Kaplan-Meier survival curves of primary tumors, recurrent tumors, and metastatic tumors that with drug treatment and those without drug treatment in each stage. The abscissa was the survival time and the ordinate was the survival rate.

Supplementary Figure S2 The distribution of patients with primary tumors, recurrent tumors and metastatic tumors at different stages in different cancer. A circle represent a type of cancer, from the inside to the outside, respectively representing stage I, stage II, stage III, and stage IV+V.

Supplementary Figure S3
**(A)**: Comparison of recurrent tumors with primary tumors, the boxplot of the expression of stage I specific differentially expressed genes. **(B)**: Comparison of metastatic tumors with primary tumors, the boxplot of the expression of stage I specific differentially expressed genes. The ordinate was the normalized gene expression value.

Supplementary Figure S4 The proportion of samples with copy number variation in primary, recurrent and metastatic patients at stage II, III, IV+V. The ordinate was the copy number variation rate, and different chromosomes were separated by dotted lines. The upper part of 0 indicated the copy number amplification rate, which was the sum of the low-level copy number amplification rate in light yellow and the high-level copy number amplification rate in dark yellow. The lower part of 0 indicated the copy number deletion rate, which was zero minus its true. The mutation rate is the sum of the single-copy deletion rate in light green and the homozygous deletion rate in dark green.

Supplementary Figure S5
**(A)**: Comparing the recurrent tumor with the primary tumor, the heatmap of log_2_FC values of genes related to four cell state. **(B)**: Comparing the metastatic tumor with the primary tumor, the heatmap of log_2_FC values of genes related to six cell state.

## References

[B1] Abbosh C. Birkbak N. J. Wilson G. A. Jamal-Hanjani M. Constantin T. Salari R. (2017). Phylogenetic ctDNA analysis depicts early-stage lung cancer evolution. Nature 545, 446–451. 10.1038/nature22364 28445469 PMC5812436

[B2] Aftimos P. Oliveira M. Irrthum A. Fumagalli D. Sotiriou C. Gal-Yam E. N. (2021). Genomic and transcriptomic analyses of breast cancer primaries and matched metastases in AURORA, the breast international group (BIG) molecular screening initiative. Cancer Discov. 11 (11), 2796–2811. 10.1158/2159-8290.CD-20-1647 34183353 PMC9414283

[B3] Anderluh M. Kocic G. Tomovic K. Kocic R. Deljanin-Ilic M. Smelcerovic A. (2016). Cross-talk between the dipeptidyl peptidase-4 and stromal cell-derived factor-1 in stem cell homing and myocardial repair: Potential impact of dipeptidyl peptidase-4 inhibitors. Pharmacol. Ther. 167, 100–107. 10.1016/j.pharmthera.2016.07.009 27484974

[B4] Aran D. Sirota M. Butte A. J. (2015). Systematic pan-cancer analysis of tumour purity. Nat. Commun. 6, 8971. 10.1038/ncomms9971 26634437 PMC4671203

[B5] Arneth B. (2019). Tumor microenvironment. Med. Kaunas. Lith. 56, 15. 10.3390/medicina56010015 PMC7023392 31906017

[B6] Bao R. Stapor D. Luke J. J. (2020). Molecular correlates and therapeutic targets in T cell-inflamed versus non-T cell-inflamed tumors across cancer types. Genome Med. 12, 90. 10.1186/s13073-020-00787-6 33106165 PMC7590690

[B7] Bhatia R. Gautam S. K. Cannon A. Thompson C. Hall B. R. Aithal A. (2019). Cancer-associated mucins: Role in immune modulation and metastasis. Cancer metastasis Rev. 38, 223–236. 10.1007/s10555-018-09775-0 30618016 PMC6614013

[B8] Bosman F. T. (1995). Prognostic value of pathological characteristics of colorectal cancer. Eur. J. cancer 31a, 1216–1221. (Oxford, England : 1990). 10.1016/0959-8049(95)00153-a 7577025

[B9] Bronkhorst A. J. Ungerer V. Holdenrieder S. (2019). Early detection of cancer using circulating tumor DNA: Biological, physiological and analytical considerations. Crit. Rev. Clin. laboratory Sci. 57, 253–269. 10.1080/10408363.2019.1700902 31865831

[B10] Cianfrocca M. Goldstein L. J. (2004). Prognostic and predictive factors in early-stage breast cancer. Oncol. 9, 606–616. 10.1634/theoncologist.9-6-606 15561805

[B11] Du J. Fu L. Ji F. Wang C. Liu S. Qiu X. (2020). FosB recruits KAT5 to potentiate the growth and metastasis of papillary thyroid cancer in a DPP4-dependent manner. Life Sci. 259, 118374. 10.1016/j.lfs.2020.118374 32891613

[B12] Fabisiewicz A. Szostakowska-Rodzos M. Zaczek A. J. Grzybowska E. A. (2020). Circulating tumor cells in early and advanced breast cancer; biology and prognostic value. Int. J. Mol. Sci. 21, 1671. 10.3390/ijms21051671 32121386 PMC7084781

[B13] Freeman H. J. (2013). Early stage colon cancer. World J. gastroenterology 19, 8468–8473. 10.3748/wjg.v19.i46.8468 PMC3870492 24379564

[B14] Gerstung M. Jolly C. Leshchiner I. Dentro S. C. Gonzalez S. Rosebrock D. (2020). The evolutionary history of 2,658 cancers. Nature 578, 122–128. 10.1038/s41586-019-1907-7 32025013 PMC7054212

[B15] Gong J. Jin S. Pan X. Wang G. Ye L. Tao H. (2018). Identification of long non-coding RNAs for predicting prognosis among patients with thymoma. Clin. Lab. 64, 1193–1198. 10.7754/Clin.Lab.2018.180136 30146820

[B16] Gouri A. Benarba B. Dekaken A. Aoures H. Benharkat S. (2020). Prediction of late recurrence and distant metastasis in early-stage breast cancer: Overview of current and emerging biomarkers. Curr. drug targets 21, 1008–1025. 10.2174/1389450121666200312105908 32164510

[B17] Hayashi N. Ito I. Yanagisawa A. Kato Y. Nakamori S. Imaoka S. (1995). Genetic diagnosis of lymph-node metastasis in colorectal cancer. Lancet (London, Engl. 345, 1257–1259. 10.1016/s0140-6736(95)90922-2 7746054

[B18] He M. Yang T. Wang Y. Wang M. Chen X. Ding D. (2021). Immune checkpoint inhibitor-based strategies for synergistic cancer therapy. Adv. Healthc. Mater. 10, e2002104. 10.1002/adhm.202002104 33709564

[B19] Hendrix A. Braems G. Bracke M. Seabra M. Gahl W. De Wever O. (2010). The secretory small GTPase Rab27B as a marker for breast cancer progression. Oncotarget 1 (4), 304–308. 10.18632/oncotarget.100809 21304180 PMC3058367

[B20] Hua R. Yu J. Yan X. Ni Q. Zhi X. Li X. (2020). Syndecan-2 in colorectal cancer plays oncogenic role via epithelial-mesenchymal transition and MAPK pathway. Biomed. Pharmacother. = Biomedecine Pharmacother. 121, 109630. 10.1016/j.biopha.2019.109630 31707342

[B21] Hwang H. S. An J. Kang H. J. Oh B. Oh Y. J. Oh J. H. (2021). Prognostic molecular indices of resectable hepatocellular carcinoma: Implications of S100P for early recurrence. Ann. Surg. Oncol. 28 (11), 6466–6478. 10.1245/s10434-021-09825-y 33786678

[B22] Jiang P. Gu S. Pan D. Fu J. Sahu A. Hu X. (2018). Signatures of T cell dysfunction and exclusion predict cancer immunotherapy response. Nat. Med. 24, 1550–1558. 10.1038/s41591-018-0136-1 30127393 PMC6487502

[B23] Jung G. T. Kim K. P. Kim K. (2020). How to interpret and integrate multi-omics data at systems level. Animal cells Syst. 24, 1–7. 10.1080/19768354.2020.1721321 PMC7048189 32158610

[B24] Khan S. Gerber D. E. (2020). Autoimmunity, checkpoint inhibitor therapy and immune-related adverse events: A review. Seminars cancer Biol. 64, 93–101. 10.1016/j.semcancer.2019.06.012 PMC6980444 31330185

[B25] Kim S. K. Kim S. Y. Kim C. W. Roh S. A. Ha Y. J. Lee J. L. (2019). A prognostic index based on an eleven gene signature to predict systemic recurrences in colorectal cancer. Exp. Mol. Med. 51, 1–12. 10.1038/s12276-019-0319-y PMC6802642 31578316

[B26] Kim M. Kwon C. H. Jang M. H. Kim J. M. Kim E. H. Jeon Y. K. (2021). Whole-exome sequencing in papillary microcarcinoma: Potential early biomarkers of lateral lymph node metastasis. Endocrinol. Metab. Seoul. 36 (5), 1086–1094. 10.3803/EnM.2021.1132 34731936 PMC8566127

[B27] Koh H. M. Song D. H. (2019). Prognostic role of Rab27A and Rab27B expression in patients with non-small cell lung carcinoma. Thorac. cancer 10, 143–149. 10.1111/1759-7714.12919 30480360 PMC6360262

[B28] Läubli H. Borsig L. (2019). Altered cell adhesion and glycosylation promote cancer immune suppression and metastasis. Front. Immunol. 10, 2120. 10.3389/fimmu.2019.02120 31552050 PMC6743365

[B29] Lee Y. T. Tan Y. J. Oon C. E. (2018). Molecular targeted therapy: Treating cancer with specificity. Eur. J. Pharmacol. 834, 188–196. 10.1016/j.ejphar.2018.07.034 30031797

[B30] Liang L. Fang J. Y. Xu J. (2016). Gastric cancer and gene copy number variation: Emerging cancer drivers for targeted therapy. Oncogene 35, 1475–1482. 10.1038/onc.2015.209 26073079

[B31] Mayekar M. K. Bivona T. G. (2017). Current landscape of targeted therapy in lung cancer. Clin. Pharmacol. Ther. 102, 757–764. 10.1002/cpt.810 28786099

[B32] Mishra S. Kaddi C. D. Wang M. D. (2016). Pan-cancer analysis for studying cancer stage using protein and gene expression data. Annu. Int. Conf. IEEE Eng. Med. Biol. Soc. 2016, 2440–2443. 10.1109/embc.2016.7591223 28268818

[B33] Nadal R. Bellmunt J. (2019). Management of metastatic bladder cancer. Cancer Treat. Rev. 76, 10–21. 10.1016/j.ctrv.2019.04.002 31030123

[B34] Ni Y. Xie G. Jia W. (2014). Metabonomics of human colorectal cancer: New approaches for early diagnosis and biomarker discovery. J. proteome Res. 13, 3857–3870. 10.1021/pr500443c 25105552

[B35] Pan H. Gray R. Braybrooke J. Davies C. Taylor C. McGale P. (2017). 20-Year risks of breast-cancer recurrence after stopping endocrine therapy at 5 years. N. Engl. J. Med. 377, 1836–1846. 10.1056/NEJMoa1701830 29117498 PMC5734609

[B36] Pascual G. Avgustinova A. Mejetta S. Martín M. Castellanos A. Attolini C. S. (2017). Targeting metastasis-initiating cells through the fatty acid receptor CD36. Nature 541, 41–45. 10.1038/nature20791 27974793

[B37] Pasechnikov V. Chukov S. Fedorov E. Kikuste I. Leja M. (2014). Gastric cancer: Prevention, screening and early diagnosis. World J. gastroenterology 20, 13842–13862. 10.3748/wjg.v20.i38.13842 PMC4194567 25320521

[B38] Qiu Y. Liu L. Yang H. Chen H. Deng Q. Xiao D. (2021). Integrating histologic and genomic characteristics to predict tumor mutation burden of early-stage non-small-cell lung cancer. Front. Oncol. 10, 608989. 10.3389/fonc.2020.608989 33996530 PMC8121003

[B39] Rueda O. M. Sammut S. J. Seoane J. A. Chin S. F. Caswell-Jin J. L. Callari M. (2019). Dynamics of breast-cancer relapse reveal late-recurring ER-positive genomic subgroups. Nature 567, 399–404. 10.1038/s41586-019-1007-8 30867590 PMC6647838

[B40] Sanchez-Vega F. Mina M. Armenia J. Chatila W. K. Luna A. La K. C. (2018). Oncogenic signaling pathways in the cancer genome atlas. Cell. 173, 321–337.e10. 10.1016/j.cell.2018.03.035 29625050 PMC6070353

[B41] Soave A. Kluwe L. Yu H. Rink M. Gild P. Vetterlein M. W. (2020). Copy number variations in primary tumor, serum and lymph node metastasis of bladder cancer patients treated with radical cystectomy. Sci. Rep. 10, 21562. 10.1038/s41598-020-75869-x 33298978 PMC7725833

[B42] Subramanian I. Verma S. Kumar S. Jere A. Anamika K. (2020). Multi-omics data integration, interpretation, and its application. Bioinforma. Biol. insights 14, 1177932219899051. 10.1177/1177932219899051 PMC7003173 32076369

[B43] Sun Y. V. Hu Y. J. (2016). Integrative analysis of multi-omics data for discovery and functional studies of complex human diseases. Adv. Genet. 93, 147–190. 10.1016/bs.adgen.2015.11.004 26915271 PMC5742494

[B44] Thorsson V. Gibbs D. L. Brown S. D. Wolf D. Bortone D. S. Ou Yang T.-H. (2019). The immune landscape of cancer. Immunity 51, 411–412. 10.1016/j.immuni.2019.08.004 31433971

[B45] Tsoyi K. Osorio J. C. Chu S. G. Fernandez I. E. De Frias S. P. Sholl L. (2019). Lung adenocarcinoma syndecan-2 potentiates cell invasiveness. Am. J. Respir. Cell. Mol. Biol. 60, 659–666. 10.1165/rcmb.2018-0118OC 30562054 PMC6543747

[B46] Wang Y. Klijn J. G. Zhang Y. Sieuwerts A. M. Look M. P. Yang F. (2005). Gene-expression profiles to predict distant metastasis of lymph-node-negative primary breast cancer. Lancet (London, Engl. 365, 671–679. 10.1016/s0140-6736(05)17947-1 15721472

[B47] Wang Q. Zhang J. Tu H. Liang D. Chang D. W. Ye Y. (2019). Soluble immune checkpoint-related proteins as predictors of tumor recurrence, survival, and T cell phenotypes in clear cell renal cell carcinoma patients. J. Immunother. cancer 7, 334. 10.1186/s40425-019-0810-y 31783776 PMC6884764

[B48] Wu G. Niu M. Qin J. Wang Y. Tian J. (2019). Inactivation of Rab27B-dependent signaling pathway by calycosin inhibits migration and invasion of ER-negative breast cancer cells. Gene 709, 48–55. 10.1016/j.gene.2019.04.005 31002890

[B49] Yang B. Zhou M. Wu Y. Ma Y. Tan Q. Yuan W. (2021). The impact of immune microenvironment on the prognosis of pancreatic ductal adenocarcinoma based on multi-omics analysis. Front. Immunol. 12, 769047. 10.3389/fimmu.2021.769047 34777388 PMC8580856

[B50] Yates L. R. Knappskog S. Wedge D. Farmery J. H. R. Gonzalez S. Martincorena I. (2017). Genomic evolution of breast cancer metastasis and relapse. Cancer Cell. 32, 169–184.e7. 10.1016/j.ccell.2017.07.005 28810143 PMC5559645

[B51] Zhai T. Muhanhali D. Jia X. Wu Z. Cai Z. Ling Y. (2019). Identification of gene co-expression modules and hub genes associated with lymph node metastasis of papillary thyroid cancer. Endocrine 66, 573–584. 10.1007/s12020-019-02021-9 31332712

[B52] Zhang W. Wang H. Sun M. Deng X. Wu X. Ma Y. (2020). CXCL5/CXCR2 axis in tumor microenvironment as potential diagnostic biomarker and therapeutic target. Cancer Commun. Lond. Engl. 40, 69–80. 10.1002/cac2.12010 PMC7163794 32237072

[B53] Zhao Y. Shao Q. Peng G. (2020). Exhaustion and senescence: Two crucial dysfunctional states of T cells in the tumor microenvironment. Cell. Mol. Immunol. 17, 27–35. 10.1038/s41423-019-0344-8 31853000 PMC6952436

[B54] Zhou H. Huang S. (2011). Role of mTOR signaling in tumor cell motility, invasion and metastasis. Curr. protein and peptide Sci. 12, 30–42. 10.2174/138920311795659407 21190521 PMC3410744

