# Supplementary figures and images for: Multi-omics analysis reveals a molecular landscape of the early recurrence and early metastasis in pan-cancer
Source: Front Genet. 2023 Apr 20;14:1061364. doi: 10.3389/fgene.2023.1061364 (PMC10157260; doi:10.3389/fgene.2023.1061364)

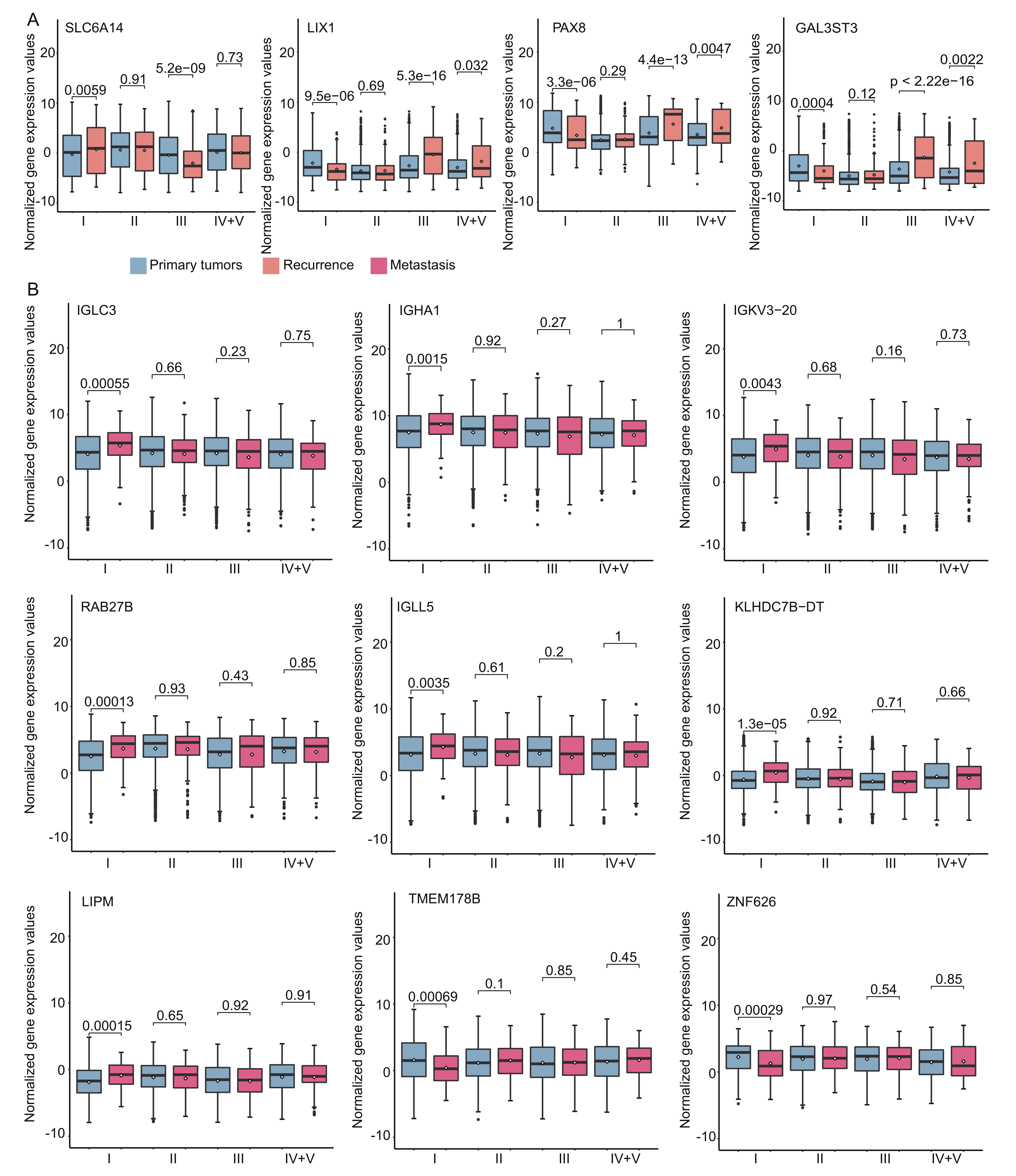

Supplement: Supplementary file 1 [file Image3.TIF]

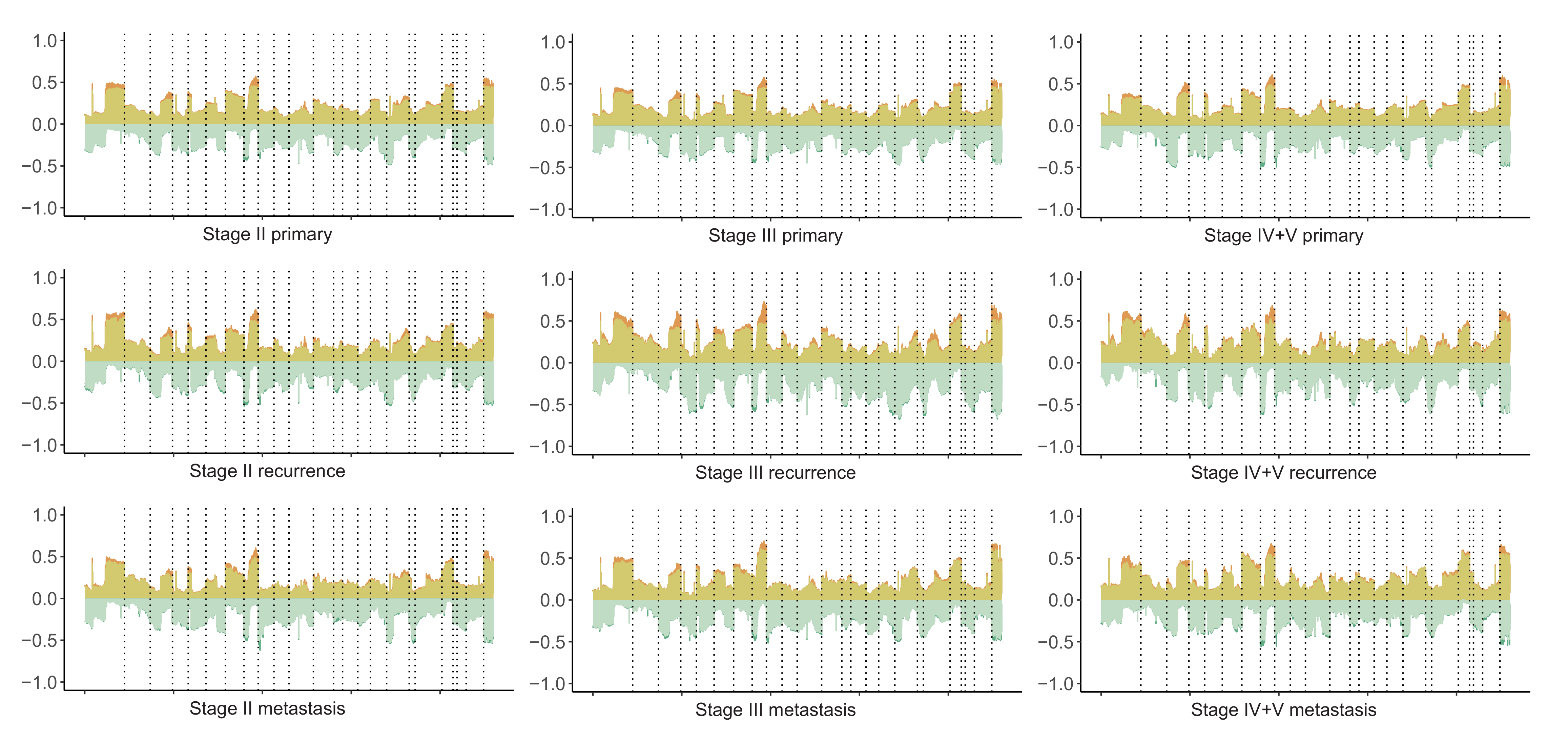

Supplement: Supplementary file 2 [file Image4.TIF]

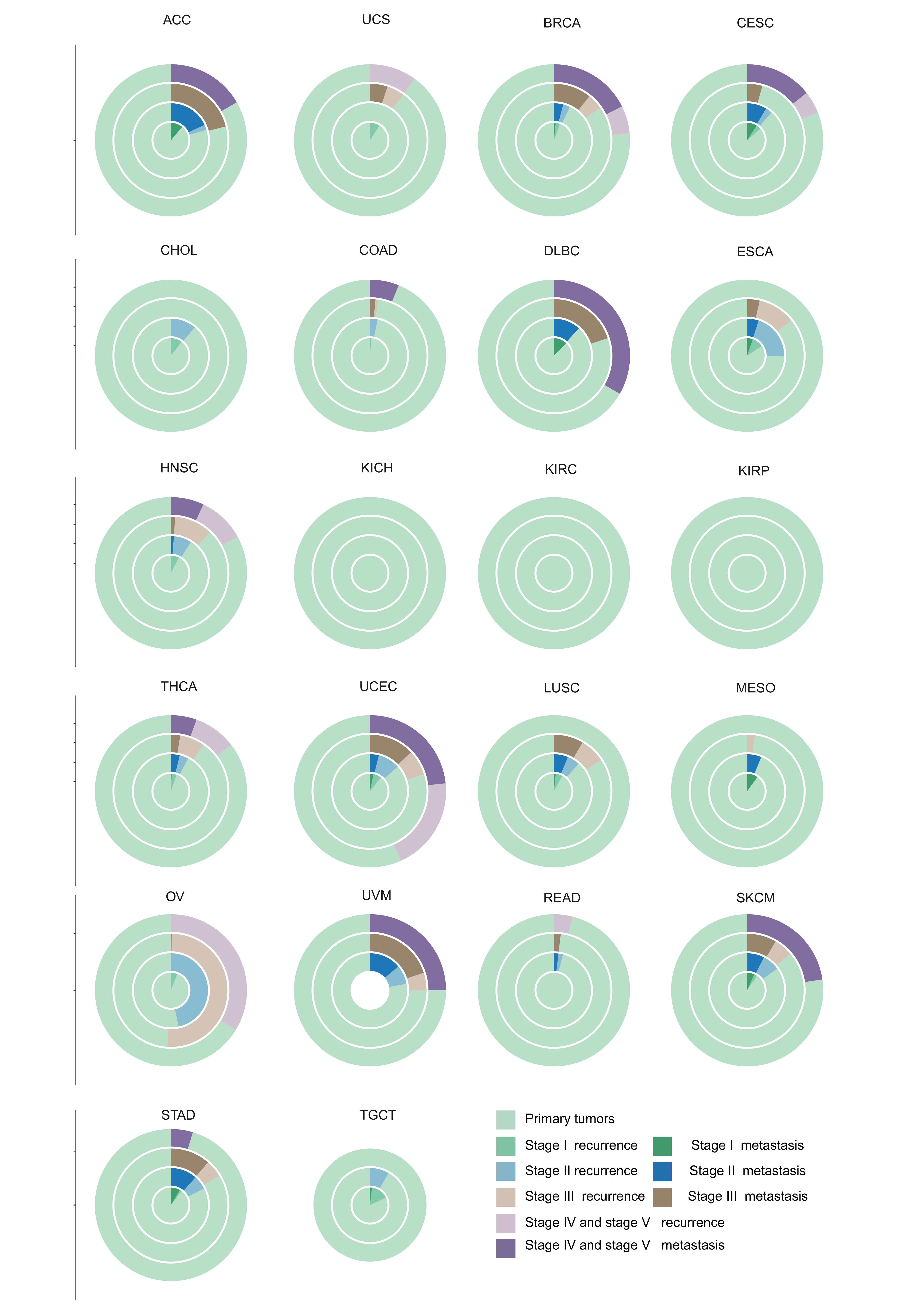

Supplement: Supplementary file 3 [file Image2.TIF]

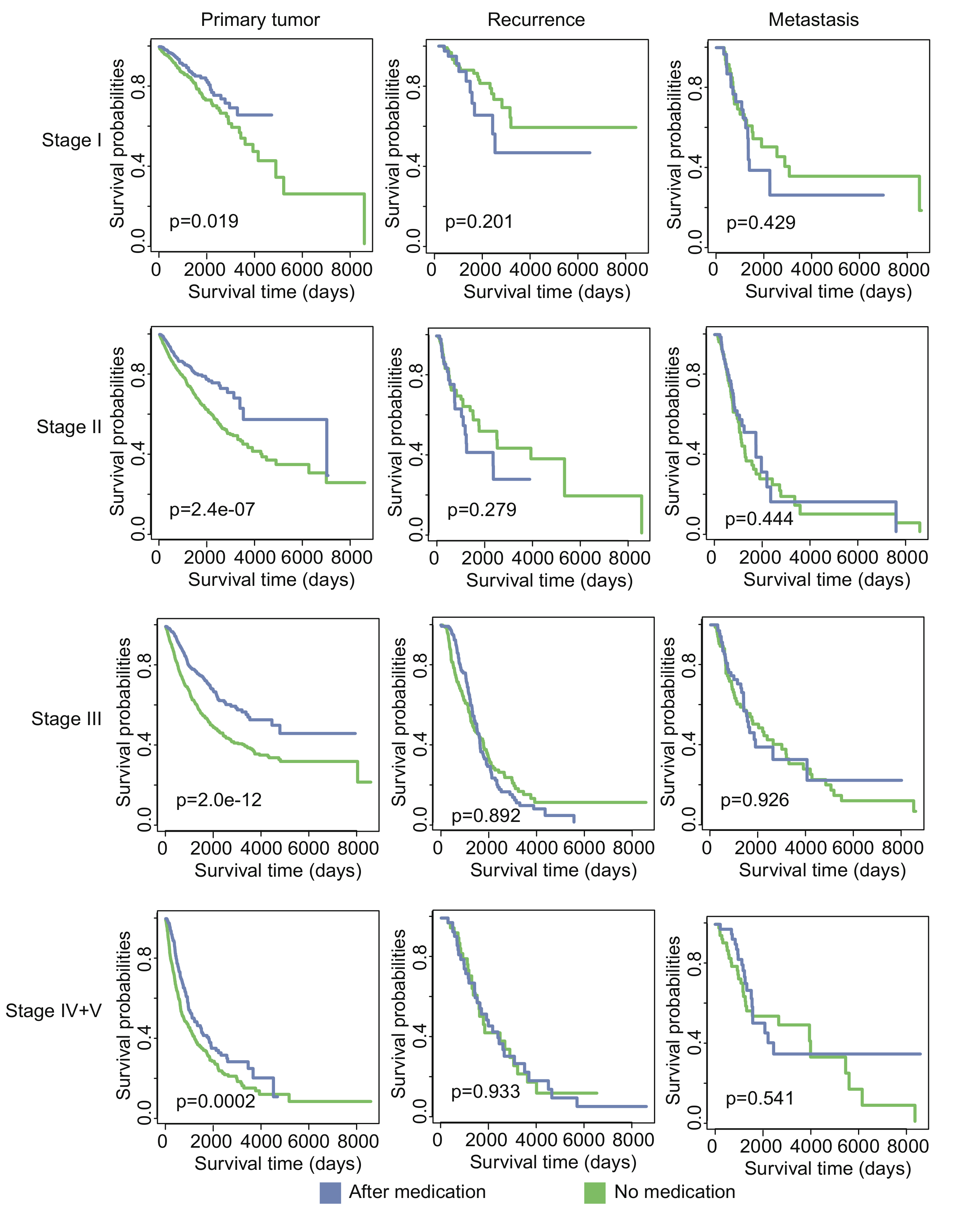

Supplement: Supplementary file 4 [file Image1.TIF]

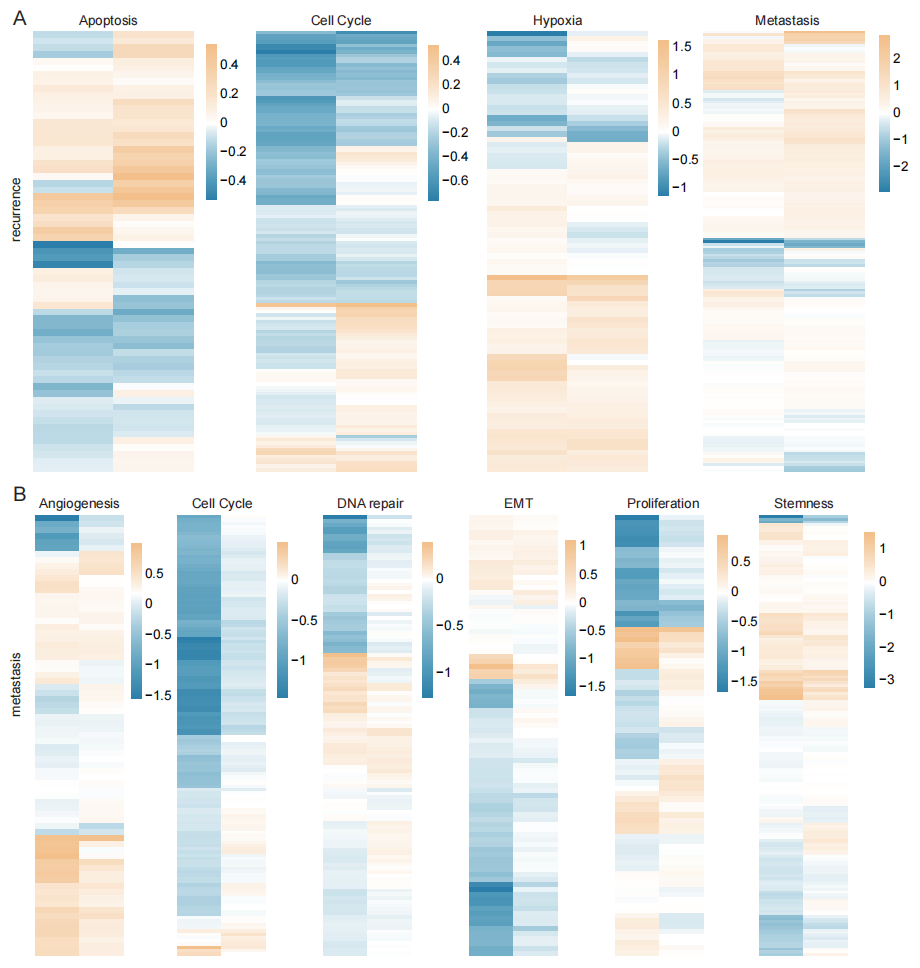

Supplement: Supplementary file 6 [file Image5.tif]
